# Esketamine alleviates the pain-depression dyad: insights from exploratory transcriptomic profiling and Wnt4-associated molecular alterations within the anterior cingulate cortex

**DOI:** 10.3389/fphar.2026.1873924

**Published:** 2026-10-16

**Authors:** Qingmei Li, Shuming Liu, Huiling Chen, Guangda Liang, Zhouyi Song, Yanfang Liu, Xingfeng Liu, Bangyong Qin

**Affiliations:** 1 Department of Anesthesiology, The Affiliated Hospital of Zunyi Medical University, Zunyi, China; 2 Department of Anesthesiology, The Affiliated Stomatological Hospital of Zunyi Medical University, Zunyi, China; 3 Guizhou Key Laboratory of Anesthesia and Organ Protection of Ministry of Education (In Cultivation), Zunyi Medical University, Zunyi, China; 4 Department of Pain Medicine, The Affiliated Hospital of Zunyi Medical University, Zunyi, China; 5 Guizhou Key Laboratory of Brain Science, Zunyi Medical University, Zunyi, China

**Keywords:** anterior cingulate cortex, esketamine, pain-depression dyad (PDD), RNA sequencing, Wnt4

## Abstract

**Objective:**

The molecular mechanisms driving esketamine (ESK) efficacy in the pain-depression dyad (PDD) remain incompletely understood. This study investigated the effects of a sub-anesthetic ESK regimen on cortical expression profiles and exploratory signaling markers within the anterior cingulate cortex (ACC).

**Methods:**

Male Sprague-Dawley rats were randomized into Sham, PDD, and PDD + ESK groups. The PDD model, established *via* chronic constriction injury (CCI), was validated using a behavioral test battery tracking mechanical allodynia and depression-like phenotypes. On day 21 post-surgery, PDD + ESK rats received a single esketamine injection (15 mg/kg, i.p.). Multi-regional c-Fos expression mapping across the PFC, HPC, and ACC identified the primary responsive structure, followed by dual immunofluorescence for cellular phenotyping. Finally, ACC excitatory neuronal calcium dynamics, exploratory transcriptomic profiles, and Wnt4-associated molecular alterations (including GSK3β and mTOR phosphorylation alongside synaptic markers) were evaluated using fiber photometry, RNA sequencing, and Western blotting.

**Results:**

ESK alleviated pain hypersensitivity, behavioral despair, and anhedonia in PDD rats. Regional c-Fos profiling identified the ACC as the primary reactive locus, with activation localized predominantly to CaMKIIα-positive neurons. Fiber photometry confirmed that ESK attenuated functional hyperactivation in these ACC excitatory neurons. Exploratory transcriptomic profiling (unadjusted *P* < 0.05) revealed candidate altered transcripts, identifying *Wnt4* as an exploratory molecular marker for further investigation. Subsequent orthogonal protein validations demonstrated that ESK modulated *Wnt4* expression alongside concomitant alterations in GSK3β and mTOR phosphorylation, parallel with the attenuation of pathologically elevated synaptic protein markers and a restorative trend in synaptic ultrastructure.

**Conclusion:**

A single sub-anesthetic ESK dose effectively alleviates PDD behavioral phenotypes in rats. This therapeutic efficacy is associated with Wnt4-related molecular adjustments coupled with putative downstream GSK3β-mTOR signaling alterations within the ACC CaMKIIα-positive neurons, providing exploratory transcriptomic and molecular insights into cortical adaptations in pain-related affective disorders.

## 1 Introduction

Neuropathic pain (NP) refers to pain arising from injury or disease of the somatosensory nervous system. Epidemiological data indicate that the prevalence of NP in the general population ranges from 3% to 17%, accounting for approximately 20%–25% of all chronic pain cases, with fewer than a quarter of patients achieving satisfactory pain relief under conventional pharmacological interventions (Baskozos et al., 2023). Accumulating clinical and preclinical evidence indicates a robust, bidirectional relationship between chronic pain and emotional affective deficits, wherein persistent pain precipitates depressive phenotypes, while depression conversely amplifies noxious perception. This mutually reinforcing pathological interaction is conceptualized as the pain-depression dyad (PDD) (Tang et al., 2022; Meda et al., 2022). Population-based surveys reveal that up to 52% of chronic pain patients exhibit comorbid affective distress, significantly complicating clinical management and exacerbating the socioeconomic burden. The anterior cingulate cortex (ACC) constitutes a critical supraspinal hub integrating nociceptive processing and emotional-affective regulation (Bliss et al., 2016). While structural and functional aberrations within the ACC are recognized components of PDD, the exact transcriptomic networks and regulatory intracellular signaling cascades driving this cortical dysfunction remain incompletely characterized.

Esketamine (ESK), a sub-anesthetic N-methyl-D-aspartate receptor (NMDAR) antagonist, exhibits rapid and sustained antidepressant efficacy, particularly in treatment-resistant depression, alongside profound analgesic properties (Yang et al., 2023; Yang et al., 2019; Elfving et al., 2022; Expert Consensus Group on Esketamine Clinical Application, 2023). However, the therapeutic mechanisms of ESK in the specific context of neuropathic pain-related depression remain insufficiently explored. Although canonical models attribute its rapid action to downstream NMDAR blockade and subsequent mechanistic target of rapamycin (mTOR) activation, growing evidence suggests that its sustained therapeutic profile involves broader homeostatic resets of upstream transcriptomic networks within pain- and emotion-integrating structures (Pisanu et al., 2025). Crucially, the precise endogenous gene programs, such as specific post-transcriptional and translational modification networks, through which ESK remodels the ACC microenvironment during comorbidity alleviation remain largely unknown (Ji et al., 2025).

The ACC is widely recognized as a pivotal substrate involved in encoding both the sensory and affective-motivational dimensions of pain (Xiao et al., 2018), with its directional projection pathways and localized subregion neural dynamics tightly controlling negative affect, cognitive control, and chronic pain-induced depressive states (Journée et al., 2026; Tolomeo et al., 2016). Peripheral nerve injury or chronic distress disrupts these localized cortical circuitries by inducing widespread molecular remodeling (Zhuo, 2019). Recent transcriptomic studies of chronic pain states have identified numerous differentially expressed genes (DEGs) within the ACC, with significant functional enrichment mapped onto calcium signaling, cholinergic synapses, axon guidance, and neuroplasticity pathways (Zhang et al., 2022; Qiu et al., 2023; Su et al., 2021). Among these interconnected networks, the wingless-type MMTV integration site family member 4 (Wnt4) and its convergent downstream intracellular branch-the glycogen synthase kinase-3 beta (GSK3β) and mTOR signaling cascade-have been increasingly implicated in the maintenance of cortical synaptic scaffolding and the regulation of homeostatic ratios between complementary excitatory and inhibitory synaptic markers. Pathological disruptions of this precise structural and translational machinery within ACC CaMKIIα-positive excitatory neurons are hypothesized to drive the neural network hyperexcitability and structural plasticity defects typically observed in depression and pain sensitization (Alexander et al., 2021; Holmes et al., 2018). Emerging clinical and preclinical evidence suggests that the robust antidepressant and emotional modulatory effects of ESK may fundamentally involve a comprehensive structural reset of these aberrant molecular pathways within pain-emotion integration structures, including distinct ACC subregions (Antos et al., 2024; Gärtner et al., 2024). However, whether ESK treatment mitigates PDD pathology by modulating ACC transcriptomic profiles and Wnt4-associated signaling components remains to be systematically investigated.

The present study utilized a chronic constriction injury (CCI)-induced rat model of PDD to systematically characterize the regulatory effects of ESK on ACC transcriptomic profiles. Through integrated RNA sequencing, differential expression profiling, functional KEGG pathway annotation, and protein-protein interaction (PPI) network topology decompositions, we aimed to map the network-level architecture of ESK treatment. Our subsequent molecular, immunofluorescence, and functional validations specifically focused on evaluating Wnt4-associated molecular alterations (including GSK3β and mTOR phosphorylation) and synaptic structural adaptations within ACC CaMKIIα-positive neurons, providing a translational framework for decoding the multi-axial therapeutic kinetics of ESK in pain-related affective disorders.

## 2 Materials and methods

### 2.1 Materials and animal ethics statement

Healthy male Sprague–Dawley rats (specific pathogen-free, aged 6–8 weeks, weighing 180–220 g) were used in this investigation. Animals were accommodated under standardized barrier facilities (22 °C ± 2 °C, 50% ± 10% humidity, and a 12 h light/dark cycle) with *ad libitum* access to water and chow. All experimental protocols were formally approved by the Animal Care and Use Committee of the Affiliated Hospital of Zunyi Medical University (Approval No. zyfy-an-2025-0694). Procedures strictly adhered to the Guide for the Care and Use of Laboratory Animals (National Research Council, 2011) and complied with the Society for Neuroscience (SfN) policies on the use of animals in neuroscience research to ensure institutional compliance and refine animal welfare. The detailed experimental allocation, sample sizes (n) for each group across various biochemical and behavioral assays, and biological replicate specifications are comprehensively summarized in Supplementary Table S1.

### 2.2 Establishment of the PDD rat model and esketamine administration

Figure 1A Surgical establishment of the CCI model was performed under intraperitoneal (i.p.) anesthesia with 1% pentobarbital sodium (40 mg/kg). Following exposure of the right sciatic nerve trunk, four loose ligatures were placed around the nerve at 1-mm intervals distal to the sciatic notch until a transient, mild twitch of the corresponding hind limb was elicited. Rats in the Sham group underwent identical surgical exposure of the sciatic nerve without ligation.

**FIGURE 1:**
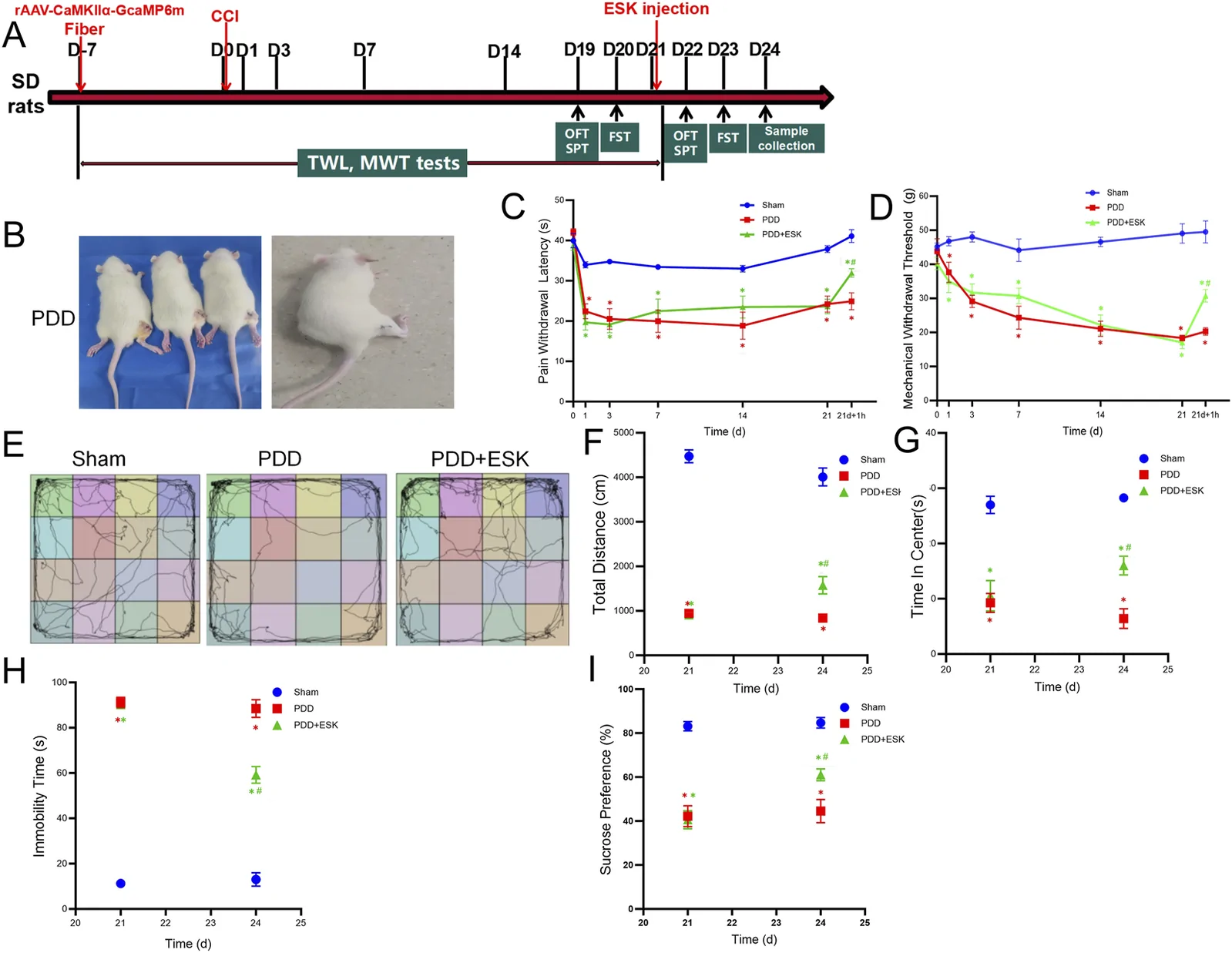
Esketamine alleviates pain-depression comorbidity in PDD model rats. **(A)** Experimental timeline and schematic flowchart of the animal model establishment, behavioral schedules, and pharmacological intervention. **(B)** Representative photographs of SD rats displaying characteristic reflexive guarding behavior and paw deformities following CCI of the sciatic nerve. **(C,D)** Temporal tracking of TWL **(C)** and MWT **(D)** across groups (n = 6 independent animals per group). Two-way repeated-measures ANOVA revealed significant main effects for TWL [Treatment: F_(2, 105)_ = 79.61, *P* < 0.0001; Time: F_(6, 105)_ = 27.40, *P* < 0.0001; Interaction:F_(12, 105)_ = 3.551, *P* = 0.0002] and MWT [Treatment:F_(2, 105)_ = 136.1, *P* < 0.0001); Time: F_(6, 105)_ = 14.52, *P* < 0.0001; Treatment: F_(12, 105)_ = 6.322, *P* < 0.0001]. **(E)** Representative horizontal movement trajectories in the open field test (OFT) on day 24. **(F,G)** Statistical quantification of total distance traveled **(F)** and time spent in the center zone **(G)** in the OFT (n = 6 independent animals per group). Two-way ANOVA showed significant differences in total distance [Treatment: F_(2, 30)_ = 353.90, *P* < 0.0001; Interaction:F_(2, 30)_ = 8.66, *P* = 0.0011; *post hoc P* = 0.0020 at D24] and center time [Treatment: F_(2, 30)_ = 64.05, *P* < 0.0001; *post hoc P* = 0.0021 at D24 for PDD vs. PDD + ESK]. **(H)** Quantitative comparison of immobility time in the forced swim test (FST) (n = 6 independent animals per group). [Treatment: F_(2, 30)_ = 434.80, *P* < 0.0001; Time: F_(1, 30)_ = 21.93, *P* < 0.0001; Interaction:F_(2, 30)_ = 20.31, *P* < 0.0001; *post hoc P* < 0.0001 at D24]. **(I)** Sucrose preference rate determined by the sucrose preference test (SPT) (n = 6 independent animals per group). [Treatment: F_(2, 30)_ = 65.62, *P* < 0.0001; Time: F_(1, 30)_ = 6.96, *P* = 0.0131; Interaction: F_(2, 30)_ = 4.01, *P* = 0.0285; *post hoc P* = 0.0116 at D24]. Data are presented as Mean ± SEM. Multiple comparisons were analyzed *via* two-way repeated-measures ANOVA (two-way RM-ANOVA) followed by Tukey’s *post hoc* test. Specific sample sizes for each assay are detailed in Supplementary Table S1 (n = 6 independent rats per group for behavioral assays **C,D,F,G,H,I **). *P < 0.05 vs. Sham; ^#^ *P* < 0.05 vs. PDD group at corresponding time points.

On post-operative day 21–a consolidated pathological window of stable pain-depression comorbidity (He et al., 2022)-pharmacological interventions were initiated. Clinical-grade esketamine hydrochloride (Jiangsu Hengrui Pharmaceuticals Co., Ltd., China) was diluted in sterile 0.9% saline to a precise working concentration of 5 mg/mL. Animals in the PDD + ESK group received a single i.p. injection of esketamine at a sub-anesthetic dose of 15 mg/kg at exactly 09:00 a.m. (Elfving et al., 2022). The Sham and PDD groups received an equivalent volume of the saline vehicle.

Downstream evaluations followed a staggered chronological schedule to isolate sustained behaviors from transient acute confounds (Figure 1A). Mechanical withdrawal threshold (MWT) and thermal withdrawal latency (TWL) were assessed during the acute phase at 1 h post-injection (10:00 a.m. on day 21). Longitudinal emotional phenotype screenings were subsequently conducted: the Sucrose Preference Test (SPT) was performed overnight across days 21–22, followed sequentially by the Open Field Test (OFT) on day 22 (24 h post-injection) and the Forced Swimming Test (FST) on day 23 (48 h post-injection).To prevent acute behavioral stress from confounding c-Fos and molecular signaling, tissue collection was performed on day 24 (72 h post-injection, ≥24 h after the final FST). Rats were euthanized under undisturbed home-cage conditions for downstream immunofluorescence, Western blotting, ELISA, and transmission electron microscopy (TEM) analyses.

### 2.3 Assessment of pain-related and depression-like behaviors

All behavioral evaluations were systematically conducted during the light cycle within a dedicated soundproof, climate-controlled testing environment (22 °C ± 1 °C).Animals were habituated to the testing apparatus for 30–60 min daily for 3 consecutive days prior to formal profiling. All behavioral tracking, filament applications, and video scoring procedures were executed by an independent investigator completely blinded to the experimental groupings.

#### 2.3.1 Mechanical nociceptive assessment

MWT was measured using an electronic von Frey anesthesiometer (Model ZS-CTY, Beijing Zhongshi Ditong Chuangke Technology Co., Ltd., Beijing, China) at baseline (day 0), on post-operative days 1, 3, 7, 10, 14, 21, and at 1 h post-injection (10:00 a.m. on day 21). Filaments were applied perpendicularly to the plantar surface of the ipsilateral right hindpaw. For each session, three independent trials were performed with a strict 5-min inter-trial interval (ITI) to prevent wind-up or sensory adaptation, and the forcing triggering brisk paw withdrawal or flinching was averaged.

#### 2.3.2 Thermal nociceptive assessment

TWL was evaluated using a radiant heat Plantar Test apparatus (Model 390, IITC Life Science, Woodland Hills, CA, United States) across identical chronologies as MWT. Following a 30-min acclimation in transparent Plexiglas chambers, a focused infrared radiant heat source was positioned beneath the center of the ipsilateral plantar surface. To prevent tissue damage, a maximum safety cutoff was fixed at 60s. Three independent trials with a 5-min ITI were utilized, and the latencies to elicit brisk withdrawal were averaged.

#### 2.3.3 Open field test

Exploratory activity and emotional reactivity were evaluated using an open field apparatus (1 m × 1 m; ZS-KC, Beijing Zhongshi Ditong Chuangke Technology Co., Ltd., China). Rats were placed in the central zone, and locomotion over a 10-min period was recorded *via* video tracking. Total travel distance and time spent in the central area were analyzed using Smart V3.0 behavioral analysis software (Panlab, Spain).

#### 2.3.4 Forced swimming test

Rats were placed in a transparent cylindrical container (diameter 20 cm, height 40 cm) to prevent the hind limbs from touching the bottom. For baseline pre-exposure, animals underwent a 10-min adaptation swim. During the formal 5 min test session, immobility duration (defined as the absence of motion except for movements necessary to keep the head above water) and immobility frequency were recorded.

#### 2.3.5 Sucrose preference test

The SPT was conducted to evaluate anhedonia. Following a 48-h habituation protocol (24 h with two bottles of 1% sucrose solution, followed by 24 h with two bottles of plain water), the formal 18-h test was initiated at 15:00 and terminated at 09:00 the following day. To eliminate side preferences, bottle positions were switched at the 9-h time point. Fluids were weighed pre- and post-test, and sucrose preference was calculated using the following formula:

Sucrose preference (%) = (sucrose solution consumption (g)/total fluid consumption (g)) × 100%

### 2.4 Glutamate quantification *via* ELISA

To determine cortical glutamate concentrations across different groups, rats were deeply anesthetized and decapitated. The ACC was rapidly microdissected on ice and cryopreserved at −80 °C for downstream assays. On the day of analysis, frozen ACC specimens werehomogenized in nine volumes of ice-cold phosphate-buffered saline (PBS, pH 7.2–7.4)using a mechanical tissue homogenizer. The crude homogenates were subsequently centrifuged at 2000–3,000 rpm for 20 min at 4 °C (TG16-W; Shanghai Luxiangyi Centrifuge Instrument Co., Ltd., China) to isolate clear supernatants.

Total glutamate (Glu) concentrations within the ACC supernatants were quantified using a commercial Rat Glu ELISA Kit (Catalog No. MM-0601R2; Jiangsu Meimian Industrial Co., Ltd., China) in strict accordance with the manufacturer’s protocols. Following standard incubation and automated multi-cycle washing sequences, horseradish peroxidase (HRP)-conjugated reagents were introduced, followed by chromogenic development utilizing tetramethylbenzidine substrate. The enzymatic reaction was ceased with a sulfuric acid stop solution, and the optical density (OD) of each well was immediately measured at a wavelength of 450 nm using a microplate reader (Infinite F50; Tecan, Switzerland). Absolute glutamate concentrations were interpolated mathematically from the standard curve, adjusted by the 5-fold sample dilution factor, and normalized against total tissue protein concentrations as determined by a standard BCA assay.

### 2.5 Transmission electron microscopy (TEM) for synaptic density

For the ultrastructural characterization of synaptic density, microdissected the ACC tissue blocks (∼1 mm^3^) were immediately immersed in 2.5% glutaraldehyde at 4 °C for 2–4 h. Following three rinses with 0.1 M phosphate buffer, specimens were post-fixed in 1% osmium tetroxide (OsO_4_) at 4 °C for 4 h. Fixed tissues were subsequently dehydrated through a graded ethanol series, infiltrated utilizing propylene oxide, and embedded in Epon-812 resin. Ultrathin sections (70 nm thickness) were sliced using a Leica UC7 ultramicrotome (Leica, Germany), collected onto 150-mesh formvar-coated copper grids, and contrast-stained with 3% uranyl acetate and 2.7% lead citrate. Microscopic examinations were executed using a JEM-1400 transmission electron microscope (JEOL, Tokyo, Japan).

Synaptic density was quantified as the number of intact synapses per unit area (number/μm^2^) based on 6 independent fields of view from 6 independent biological replicates per experimental group (n = 6 fields from N = 6 rats). Morphological identification of an intact synapse strictly required the concurrent visualization of clustered presynaptic vesicles, a distinct synaptic cleft, and a prominent post-synaptic density (PSD) profile within the same field of view. Both image acquisition and downstream synaptic tracking were executed by an independent investigator blinded to the experimental groupings.

### 2.6 *In vivo* fiber photometry for calcium recording and data analysis

#### 2.6.1 Stereotaxic surgery and viral infusion

Rats were anesthetized and secured in a stereotaxic frame. To record calcium signals specifically within the excitatory neurons of the ACC, an adeno-associated virus expressing a calcium indicator under the control of a cell-type-specific promoter (rAAV2/9-CaMKIIα-GCaMP6m; titer ≥5.00 × 10^12^ VG/mL; Brain Case, Wuhan, China) was microinjected into the ACC (500 nL). The stereotaxic coordinates were designated as: anterior-posterior (AP) +1.30 mm, medial-lateral (ML) ±0.55 mm (unilateral), and dorsal-ventral (DV) −2.8 mm relative to Bregma. Following viral infusion, a low-autofluorescence ceramic optical fiber ferrule (200 μm core diameter, NA = 0.37) was chronically implanted approximately 0.1 mm above the injection site and permanently anchored to the skull using dental cement. Animals recovered for at least 3 weeks to allow robust viral expression.

#### 2.6.2 Signal recording and mechanical stimulation

Rats were connected to a multi-channel fiber photometry system (Inper, China) *via* a 1-m single-channel low-autofluorescence fiber patch cord and habituated to the tethering environment for 3 consecutive days prior to formal data acquisition on post-operative day 21. Modulated excitation lights were delivered continuously at a sampling rate of 50 Hz: a 470 nm LED channel (calcium-dependent GCaMP6m fluorescence, power <30 μW) and a 410 nm LED isosbestic reference channel (to eliminate motion and photobeaching artifacts).To track evoked responses, calcium transients were recorded surrounding a mechanical stimulation challenge applied *via* a 6 g von Frey filament to the ipsilateral hindpaw plantar surface for a 60 s window. Each session comprised a 20-s pre-stimulus baseline period (from −20 s to 0 s) and a 60-s post-stimulus recording period (from 0 s to 60 s).

#### 2.6.3 Data processing and metric quantification

Raw fluorescence data were processed using Inper Plot software (Version 1.8.0, Inper, Hangzhou, China). Baseline correction was computed *via* a second-order polynomial fitting regression model to eliminate systemic photobleaching. The baseline-corrected relative change in calcium fluorescence was quantified using the standard baseline-subtraction algorithm: ΔF/F= (F_470_ - F_fitted 410_)/F_fitted 410_, where F470 represents the real-time green channel intensity and Ffitted410 denotes the fitted baseline reference. To normalize signal variances across multiple animals, the ΔF/F values were converted into standard Z-score values: Z-score = (ΔF/F - μ_baseline_)/σ_baseline_, where μ_baseline_ and σ_baseline_ represent the mean and standard deviation of the fluorescence signal calculated within the 20-s pre-stimulus baseline window, respectively. Evoked responses during the 60-s mechanical stimulation window were quantified by calculating the integrated area under the curve (Z-score AUC) and the peak amplitude (Peak).Following behavioral sessions, rats were sacrificed to histologically confirm precise fiber tract localization and robust GCaMP6m expression within the targeted ACC. Animals were excluded from the final analysis based on pre-established criteria: (1) optical cannula loosening or detachment during recording sessions, (2) excessive motion artifacts compromising the signal-to-noise ratio, or (3) off-target fiber placement confirmed by *post hoc* histological verification. Consequently, a balanced cohort of n = 5 animals per experimental group (Sham, PDD, and PDD + ESK) meeting these inclusion criteria was included for quantitative analysis.

### 2.7 Exploratory transcriptomic and bioinformatic analyses

#### 2.7.1 RNA isolation, library preparation, and sequencing

Rats were sacrificed under deep anesthesia, and ACC tissues were rapidly dissected, snap-frozen on dry ice, and stored at −80 °C (n = 5 per group, total N = 15). Total RNA isolation, complementary DNA (cDNA) library construction, and high-throughput sequencing were performed by Shanghai Majorbio Bio-pharm Technology Co., Ltd. RNA concentration and purity were measured using a NanoDrop 2000 spectrophotometer (Thermo Fisher Scientific, United States), and RNA integrity was validated using an Agilent 5,300 Fragment Analyzer (Agilent Technologies, United States). All samples strictly met the quality threshold of RNA Quality Number (RQN) > 6.5. Poly (A) mRNA was selectively enriched, fragmented, and reverse-transcribed into cDNA libraries according to standard Illumina protocols. High-throughput paired-end sequencing was executed on an Illumina NovaSeq platform, generating at least 5.9 Gb of clean data per sample.

#### 2.7.2 Read mapping, quantification, and differential expression

Raw sequencing reads were pre-processed using fastp software (version 0.23.4) for quality trimming and adapter removal. The resulting high-quality clean reads were aligned to the *Rattus norvegicus* reference genome (assembly mRatBN7.2) utilizing HISAT2 (version 2.2.1). Gene expression levels and read counts were quantified using RSEM (version 1.3.3). Differential expression analysis between experimental groups was carried out using the DESeq2 R package (version 1.42.0). Given the exploratory, hypothesis-generating objective of the transcriptomic profiling, candidate differentially expressed transcripts were identified based on nominal thresholds of ∣log_2_ fold change∣≥ 1 and an unadjusted *P*-value <0.05. Both unadjusted *P*-values and Benjamini–Hochberg false discovery rate (FDR) adjusted *P*-values (*P*_adjust_) were calculated and transparently reported. We explicitly acknowledge that individual transcripts did not survive standard genome-wide FDR correction (*P*_adjust_ > 0.05); therefore, transcriptomic leads were treated strictly as exploratory observations prioritized for subsequent orthogonal validation at the protein level.

#### 2.7.3 Functional enrichment and network analysis

Gene Ontology (GO) and Kyoto Encyclopedia of Genes and Genomes (KEGG) pathway enrichment analyses were conducted using goatools (version 1.4.4) and the KEGG database (version 2023.09), respectively.Statistical thresholds for functional enrichment were set at an exploratory unadjusted *P*-value <0.05 to facilitate the detection of subtle, coordinated pathway fluctuations within the ACC microenvironment without imposing rigid *post hoc* statistical exclusions. Protein–protein interaction (PPI) networks were standardly constructed utilizing the STRING database (version V12.0). All downstream bioinformatics analyses were synchronously supported by the Majorbio Cloud platform (www.majorbio.com) (Han et al., 2024).The raw sequencing datasets generated during the current study have been deposited in the NCBI Gene Expression Omnibus (GEO) database and are publicly accessible under the accession number GSE330695.

### 2.8 Reverse transcription-quantitative polymerase chain reaction (RT-qPCR)

To validate the key candidate genes identified from transcriptomic sequencing (including *Wnt4, Chrna4, Prkcd, and Ntng1*), total RNA was isolated from ACC tissue specimens (n = 3 per group) using the TRIpure Total RNA Extraction Reagent (ELK Biotechnology, China). Reverse transcription was subsequently performed to synthesize first-strand cDNA utilizing the EntiLink first Strand cDNA Synthesis Kit (ELK Biotechnology, China). Real-time quantitative PCR amplification was performed on a StepOne Real-Time PCR system (Life Technologies, United States) using the EnTurbo SYBR Green PCR SuperMix kit (ELK Biotechnology, China).

The thermal cycling parameters consisted of an initial denaturation step at 95 °C for 3 min, followed by 40 cycles of denaturation at 95 °C for 10 s, annealing at 58 °C for 30 s, and extension at 72 °C for 30 s. Amplification specificity was *post hoc* verified *via* melting curve analysis. All reactions for each biological sample were performed in technical triplicates. Amplification efficiencies for all primer pairs were validated within the standard range of 95%–105%. Detailed nucleotide sequences and primer parameters are structured in Supplementary Table S2. Relative mRNA expression levels of candidate genes were quantified using the comparative 2^−ΔΔCT^ method, with *Gapdh s*erving as the internal reference gene for normalization.

### 2.9 Western blot analysis

#### 2.9.1 Protein extraction and sample preparation

Following behavioral testing, rats were deeply anesthetized and decapitated; the whole brain was rapidly harvested on ice. ACC tissues were precisely microdissected guided by a rat stereotaxic atlas, weighed, snap-frozen in liquid nitrogen, and stored at −80 °C. Tissues were homogenized on ice in RIPA lysis buffer supplemented with protease and phosphatase inhibitor cocktails (Coolaber, China). After centrifugation at 12,000 g for 10 min at 4 °C, the supernatants were collected and protein concentrations were standardly determined *via* the BCA method (Coolaber, China).Equal amounts of protein samples (20 μg per sample) were mixed with 5× loading buffer, denatured at 95 °C for 10 min, separated by 8%–12% SDS-PAGE electrophoresis (Coolaber, China), and electro-transferred onto PVDF membranes (Millipore, United States). Membranes were blocked with 5% non-fat milk or 5% bovine serum albumin (BSA) in TBST for 2 h at room temperature.

#### 2.9.2 Antibody incubation and chemiluminescent detection

Membranes were incubated overnight at 4 °C with the following specific primary antibodies: rabbit anti-c-Fos (1:1,000, Zen-Bioscience, #340249), rabbit anti-Wnt4 (1:1,000, Zen-Bioscience, #680852), rabbit anti-P-mTOR (Ser2448) (1:1,000, Zen-Bioscience, #381557), rabbit anti-mTOR (1:1,000, Zen-Bioscience, #R380411), rabbit anti-P-4EBP1 (Thr37) (1:1,000, Zen-Bioscience, #310224), rabbit anti-4EBP1 (1:1,000, Zen-Bioscience, #R24197), rabbit anti-P-GSK3β (Ser9) (1:1,000, Zen-Bioscience, #310010), rabbit anti-GSK3β (1:1,000, Zen-Bioscience, #340449), rabbit anti-GluA1 (1:1,000, Zen-Bioscience, #R381355), rabbit anti-PSD95 (1:1,000, Zen-Bioscience, #R381001), mouse anti-GAD67 (1:1,000, Proteintech, #67648-1-Ig), mouse anti-CaMKIIα (1:2000, Proteintech, #66843-1-Ig), rabbit anti-HSP90 (1:2,000, Zen-Bioscience, #R26871), HRP-conjugated rabbit anti-β-tubulin monoclonal antibody (1:10,000, HUABIO, #HA724236H), and either mouse anti-GAPDH (1:5,000, Zen-Bioscience, #250133) or recombinant rabbit anti-GAPDH (1:5,000, Proteintech, #81640-5-RR) as the internal loading controls, which were selectively utilized and matched based on the host species of the respective proteins of interest.

Following three 5-min washes in TBST, membranes were incubated with HRP-conjugated secondary antibodies (1:5,000, Proteintech) at room temperature for 2 h. Protein bands were visualized with enhanced chemiluminescence (ECL) reagents (Millipore, United States) and quantified densitometrically *via* ImageJ software. Relative expression metrics were calculated as the optical density ratio of each evaluated protein (including both phosphorylated and total isoforms) normalized to its corresponding matched internal control (GAPDH or HSP90).

### 2.10 Immunofluorescence staining

#### 2.10.1 Tissue preparation and cryosectioning

Rats were deeply anesthetized and transcardially perfused with ice-cold saline, followed by fixation with 4% paraformaldehyde (PFA) in PBS. Brains were harvested, post-fixed in 4% paraformaldehyde (PFA) at 4 °C for 24 h, and cryoprotected in a 30% sucrose solution until completely submerged. Coronal sections (30 μm thick), primarily focusing on ACC, prefrontal cortex (PFC), and hippocampus (HPC) were precisely sliced using a cryostat based on a rat stereotaxic atlas.

#### 2.10.2 Antibody incubation and image acquisition

Free-floating sections were washed with PBS, permeated and blocked with a blocking solution (5% BSA +0.3% Triton X-100 in PBS) for 2 h at room temperature. Sections were subsequently incubated overnight at 4 °C with specific primary antibodies or cocktails: rabbit anti-wnt4 (1:200, Abcam, #ab232801), rabbit anti-c-Fos (1:200, Zen-Bioscience, #340249), mouse anti-GAD67 (1:500, Proteintech, #67648-1-Ig), and mouse anti-CaMKIIα (1:500, Proteintech, #66843-1-Ig).

After thorough washing, sections were incubated with corresponding fluorophore-conjugated secondary antibodies (Goat Anti-Rabbit IgG H&L Alexa Fluor 594, #550043; Goat Anti-Rabbit IgG H&L Alexa Fluor 488, #550037; Goat Anti-Mouse IgG H&L Alexa Fluor 488, #550036; Goat Anti-Mouse IgG H&L Alexa Fluor 594, #550042; or Goat Anti-Rabbit IgG H&L Cy5.5, 1:500, Zen-Bioscience) for 2 h at room temperature in the dark. Sections were mounted onto glass slides and coverslipped with anti-fade mounting medium containing DAPI. Digital panoramic images were captured utilizing a V200 digital slide scanner (3DHISTECH, Budapest, Hungary) and processed *via* CaseViewer software. The far-red fluorescent channel for Cy5.5-labeled c-Fos was pseudo-colored as magenta for multi-channel visualization.

#### 2.10.3 Quantification

For metric quantification, 3–5 representative, non-overlapping fields of view per animal were sampled within the specific subregions. The mean fluorescence intensity (MFI) and cross-sectional intensity line-scan distribution profiles were quantitatively analyzed using ImageJ software by an investigator blinded to the experimental groupings.

### 2.11 Statistical analysis

Data are presented as mean ± SEM. All statistical analyses and graphical representations were performed using GraphPad Prism software (version 10.0). For longitudinal behavioral data involving repeated measurements over time (e.g., MWT and TWL), two-way repeated-measures analysis of variance (two-way RM-ANOVA) was performed with Treatment Group and Time as factors, followed by Tukey’s *post hoc* test for multiple comparisons. For endpoint molecular and histological data measured at a single time point, normality was assessed using the Shapiro-Wilk test. Group comparisons were evaluated using one-way analysis of variance (ANOVA) followed by Tukey’s *post hoc* test for normally distributed data; otherwise, the Kruskal–Wallis test followed by Dunn’s *post hoc* test was applied. A two-tailed *P*-value <0.05 was considered statistically significant.

## 3 Results

### 3.1 Characterization of the PDD model and the therapeutic efficacy of esketamine

To confirm the successful establishment of the PDD model, behavioral assessments were tracked continuously for 21 days following CCI. Relative to the Sham group, PDD model rats exhibited characteristic nociceptive behaviors, including right hind limb flexion and guarding (Figure 1B). In parallel, longitudinal tracking showed that TWL and MWT were markedly reduced over the 21-day modeling period (Figures 1C,D). Concurrently, during the pre-injection baseline screenings, the modeling cohort displayed significant depression-like phenotypes on days 19 and 20, characterized by decreased total locomotor distance and reduced time spent in the central zone during the OFT, a diminished sucrose preference index, and a prolonged immobility time in the FST (Figures 1F–I). These findings confirm that by day 21 after CCI, the rats developed robust mechanical and thermal hyperalgesia tightly coupled with stable depression-like behaviors.

Following a single sub-anesthetic administration of esketamine (15 mg/kg) on day 21, distinct acute and sustained therapeutic profiles were observed. During the acute phase at 1 h post-injection, nociceptive thresholds increased and thermal/mechanical hyperalgesia were alleviated (Figures 1C,D). During the habituation window at this 1-h post-injection mark, the animals exhibited intact righting reflexes and active grid engagement, ruling out gross pharmacological sedation or motor deficits.

This acute anti-nociceptive phase transitioned into sustained mechanical and emotional recovery during the post-acute windows when active drug residues were cleared. By day 24 (72 h post-injection), esketamine-treated rats demonstrated an attenuation of PDD-induced deficits: total locomotor activity and central-zone residence time in the OFT increased (Figures 1F,G), sucrose preference was restored (Figure 1I), and immobility time in the FST was reduced (Figure 1H), validating sustained antidepressant-like efficacy. Importantly, because OFT ambulation may be influenced by pain alleviation, the concurrent improvements in the non-locomotor SPT and FST indicate that ESK relieves depressive-like deficits independently of its anti-allodynic effect.

### 3.2 Esketamine modulates abnormal neuronal activity and synaptic remodeling in the ACC of PDD

To identify brain regions exhibiting abnormal neuronal activation in the PDD model, we compared c-Fos expression in the anterior cingulate cortex (ACC), prefrontal cortex (PFC), and hippocampus (HPC) (Figure 2A). The ACC showed a significant increase in the density of c-Fos-positive neurons, indicating localized cellular hyperreactivity within this region (Figure 2B). Consistent with these histological changes, Western blot analysis confirmed a marked upregulation of c-Fos protein expression in the ACC of PDD rats, which was effectively attenuated by esketamine (ESK) treatment (Figures 2C,D). Co-immunostaining accompanied by fluorescence intensity line-scan profiles qualitatively illustrated spatial signal overlap of c-Fos within representative CaMKIIα-positive glutamatergic neurons and GAD67-positive GABAergic neurons (Figures 2E–G). Consistently, ELISA analysis demonstrated a significant elevation of glutamate levels in the ACC (Figure 2H).

**FIGURE 2:**
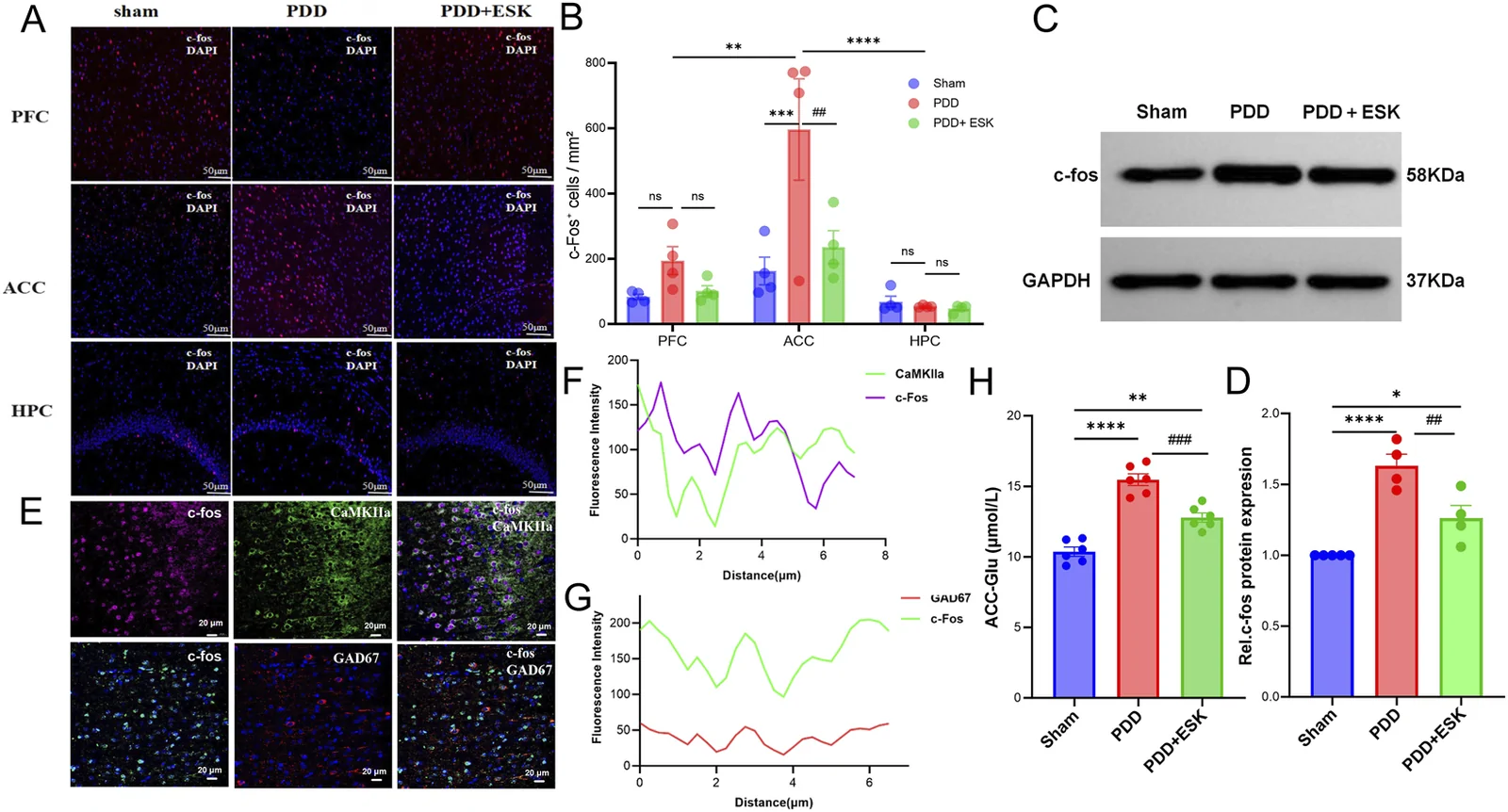
Effects of esketamine on cell-type-specific c-Fos activation and glutamate levels in the ACC of PDD model rats. **(A)** Representative immunofluorescence images showing c-Fos expression (red) co-stained with DAPI (blue) in the prefrontal cortex (PFC), anterior cingulate cortex (ACC), and hippocampus (HPC). Scale bars = 50 μm. **(B)** Statistical quantification of c-Fos^+^ cell density (cells/mm^2^) across different brain regions (n = 4 independent animals per group). **(C,D)** Representative Western blot bands **(C)** and quantitative analysis **(D)** of relative c-Fos protein expression in the ACC, with GAPDH used as the internal loading control (*N* = 4 independent animals per group). **(E)** Representative double-immunofluorescence staining images in the ACC (Scale bars = 20 μm). Top row: c-Fos (purple/pseudo-colored magenta) co-stained with the glutamatergic marker CaMKIIα (green). Bottom row: c-Fos (green) co-stained with the GABAergic marker GAD67 (red). **(F,G)** Line-scan co-localization curve profiles showing the normalized fluorescence intensity along selected linear trajectories for c-Fos and CaMKIIα **(F)**, and c-Fos and GAD67 **(G)**. **(H)** Quantification of glutamate (Glu) concentration (μmol/L) in the ACC across groups (N = 6 independent biological replicates per group). Data are presented as Mean ± SEM. Statistical analysis for **(B)** was performed using two-way ANOVA followed by Tukey’s *post hoc* test (ACC: PDD vs. Sham, ***P = 0.0005; PDD + ESK vs. PDD, ^##^ *P* = 0.0048; PDD inter-region: ACC vs. PFC, **P = 0.0013; ACC vs. HPC, ****P < 0.0001). Statistical analyses for **(D,H)** were performed *via* one-way ANOVA followed by Tukey’s *post hoc* test (**D**: PDD vs. Sham, ****P < 0.0001; PDD + ESK vs. PDD, ^##^ *P* = 0.0070; Sham vs. PDD + ESK, *P = 0.0354; **(H)** PDD vs. Sham, ****P < 0.0001; PDD + ESK vs. PDD, ^###^ *P* = 0.0006; Sham vs. PDD + ESK, *P = 0.0013),ns, no significance (*P* > 0.05).

ACC structural and functional dysregulation was subsequently assessed. Transmission electron microscopy revealed significantly reduced synaptic density in PDD rats, indicating impaired structural connectivity (Figures 3A,B). Paradoxically, real-time fiber photometry showed that despite this structural loss, ACC excitatory neurons in the PDD group exhibited enhanced and prolonged calcium transients under noxious mechanical stimulation (von Frey filament, 6 g, 60 s), characterized by a significant increase in both the Z-score area under the curve (AUC) and peak amplitude compared to the Sham group (Figures 3C–G). ESK administration suppressed this functional hyperactivity, with a trend toward improving synaptic density (although not statistically significant, Figure 3B), suggesting that ESK may alleviate PDD-like behaviors predominantly by rectifying aberrant functional hyperactivity.

**FIGURE 3:**
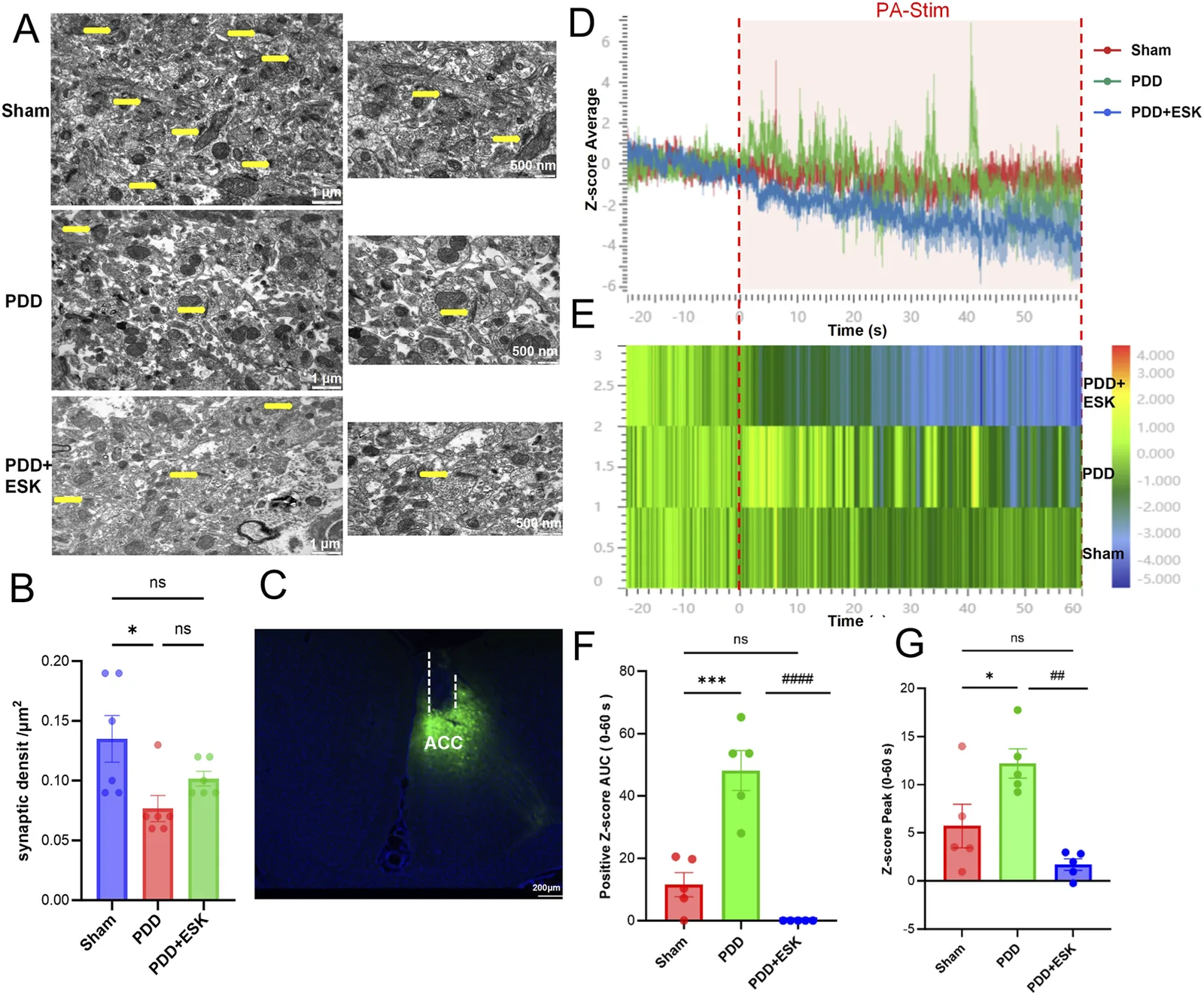
Esketamine mitigates synaptic impairment and attenuates neuronal calcium hyperactivation in the ACC of PDD rats. **(A)** Representative transmission electron microscopy (TEM) micrographs highlighting synaptic ultrastructure within the specific ACC layers across experimental groups (left panels, scale bars = 1 μm; right panels, high-magnification inserts, scale bars = 500 nm). Intact synapses (yellow horizontal bars) were morphologically identified by clustered presynaptic vesicles, a distinct synaptic cleft, and a prominent postsynaptic density (PSD). **(B)** Statistical quantification of synaptic density (synapses/μm2; n = 6 independent animals per group) analyzed *via* one-way ANOVA followed by Tukey’s *post hoc* test (*P = 0.0209 for PDD vs. Sham; P > 0.05, ns, for PDD vs. PDD + ESK). **(C)** Histological and fluorescence microscopic verification of rAAV2/9-CaMKIIα-GCaMP6m expression and precise fiber-optic cannula tract localization above the investigated ACC layer. **(D)** Time-course representative calcium fluorescence signal traces (Positive Z-score average) of ACC excitatory neurons in response to a sustained mechanical stimulation challenge (6 g von Frey filament applied to the hindpaw for a continuous 60 s window, indicated by the red shaded PA-Stim area). **(E)** Corresponding representative fluorescence intensity heatmaps tracking real-time calcium population transients throughout the pre-stimulus baseline (−20 s–0 s) and stimulus recording (0 s–60 s) phases across groups. **(F,G)** Statistical quantification of the integrated area under the curve (Positive Z-score AUC) **(F)** and peak amplitude (Z-score Peak) **(G)** during the 60-s mechanical stimulation window. One-way ANOVA followed by Tukey’s *post hoc* test revealed a marked increase in evoked calcium influx in the PDD rats [**F** (AUC): ***P = 0.0002 vs. Sham, ^####^ *P* < 0.0001 vs. PDD; **G** (Peak): *P = 0.0361 vs. Sham, ^##^ *P* = 0.0016 vs. PDD]. Data are presented as Mean ± SEM. Multiple comparisons were evaluated *via* one-way ANOVA followed by Tukey’s *post hoc* test. Specific sample sizes (*n* = 6 for TEM assay B; *n* = 5 for calcium photometry assays **(F,G)** are aligned with Supplementary Table S1. *P < 0.05, ***P < 0.001 vs. Sham group; ^##^ *P* < 0.01, ^####^ *P* < 0.0001 vs. PDD group; ns, not significant.

### 3.3 Exploratory transcriptomic profiling of the ACC across groups

#### 3.3.1 Exploratory analysis of candidate differentially expressed transcripts

RNA sequencing was performed on ACC tissues from three groups (*n* = 5 per group), yielding 15 transcriptomic datasets. A total of 20,776 expressed genes and 39,759 transcripts were identified. Under standard multiple-testing correction (Benjamini–Hochberg FDR), no transcripts met genome-wide statistical significance (*P*_adjust_ > 0.05, Table 1). Thus, prioritizing hypothesis generation, candidate differentially expressed genes (candidate DEGs) were screened using nominal exploratory thresholds (∣log_2_ Fold Change∣≥ 1.0, unadjusted *P* < 0.05). This yielded 124 candidate DEGs in PDD vs. Sham (90 upregulated, 34 downregulated), and 83 DEGs in PDD + ESK vs. PDD (38 upregulated, 45 downregulated) (Figures 4A–C), serving as exploratory leads for subsequent orthogonal validation.

**TABLE 1:** Seventeen overlapping genes from transcriptomic comparisons.

Gene name	Gene description	PDD vs. Sham log_2_ FC	PDD vs. Sham *P*-value	PDD vs. Sham *P* adjust	PDD + ESK vs. PDD log_2_ FC	PDD+ESK vs. PDD *P*-value	PDD + ESKvs. PDD *P* adjust
*Eddm3b*	epididymal protein 3B	3.05	0.0264	0.9988	−3.69	0.0114	0.9999
*RGD1561442*	similar to Vinculin (Metavinculin)	−1.71	0.0203	0.9988	2.11	0.0016	0.9999
*Prkcd*	protein kinase C, delta	1.17	0.0267	0.9988	−1.28	0.0135	0.9999
*Col26a1*	collagen type XXVI alpha 1 chain	3.29	0.0094	0.9988	−2.82	0.0216	0.9999
*Steap4*	STEAP4 metalloreductase	−1.33	0.0271	0.9988	1.51	6.40*10−^5^	0.2437
*Wnt4*	Wnt family member 4	1.34	0.0115	0.9988	−1.21	0.0148	0.9999
*Atp2a1*	ATPase sarcoplasmic/endoplasmic reticulum Ca2+ transporting 1	1.07	0.0158	0.9988	−1.61	0.0002	0.5605
*Bricd5*	BRICHOS domain containing 5	−1.18	0.0243	0.9988	1.11	0.0327	0.9999
*Eno1ps1*	enolase 1, pseudogene 1	4.33	0.0011	0.6571	−3.24	0.0124	0.9999
*Kcne2*	potassium voltage-gated channel subfamily E regulatory subunit 2	1.32	0.0061	0.9988	−1.07	0.0193	0.9999
*Siglec8*	sialic acid binding Ig-like lectin 8	2.43	0.0027	0.9362	−1.84	0.0229	0.9999
*Sult1c2a*	sulfotransferase family 1C member 2A	−2.41	0.0207	0.9988	2.47	0.0139	0.9999
*Ptpn3*	protein tyrosine phosphatase, non-receptor type 3	2.59	0.0099	0.9988	−2.76	0.0028	0.9999
*LOC100912568*	probable ATP-dependent RNA helicase DDX41-like	−2.26	0.0118	0.9988	2.69	0.0009	0.9999
*Ntng1*	netrin G1	1.06	0.0463	0.9988	−1.26	0.0113	0.9999
*Chrna4*	cholinergic receptor nicotinic alpha 4 subunit	1.14	0.0228	0.9988	−1.09	0.0287	0.9999
*Styk1*	serine/threonine/tyrosine kinase 1	3.79	0.0226	0.9988	−3.86	0.0179	0.9999

DEGs, differentially expressed genes; PDD, pain-depression comorbidity; ESK, esketamine. n = 5 independent biological samples per group. Unadjusted *P*-value and Benjamini-Hochberg adjusted *P*-value (Padjust) were calculated using DESeq2. An unadjusted *P*-value < 0.05 and |log_2_ FC| ≥1.0 served as an exploratory screening threshold for DEGs, with selected candidate targets independently validated by RT-qPCR and Western blotting.

**FIGURE 4:**
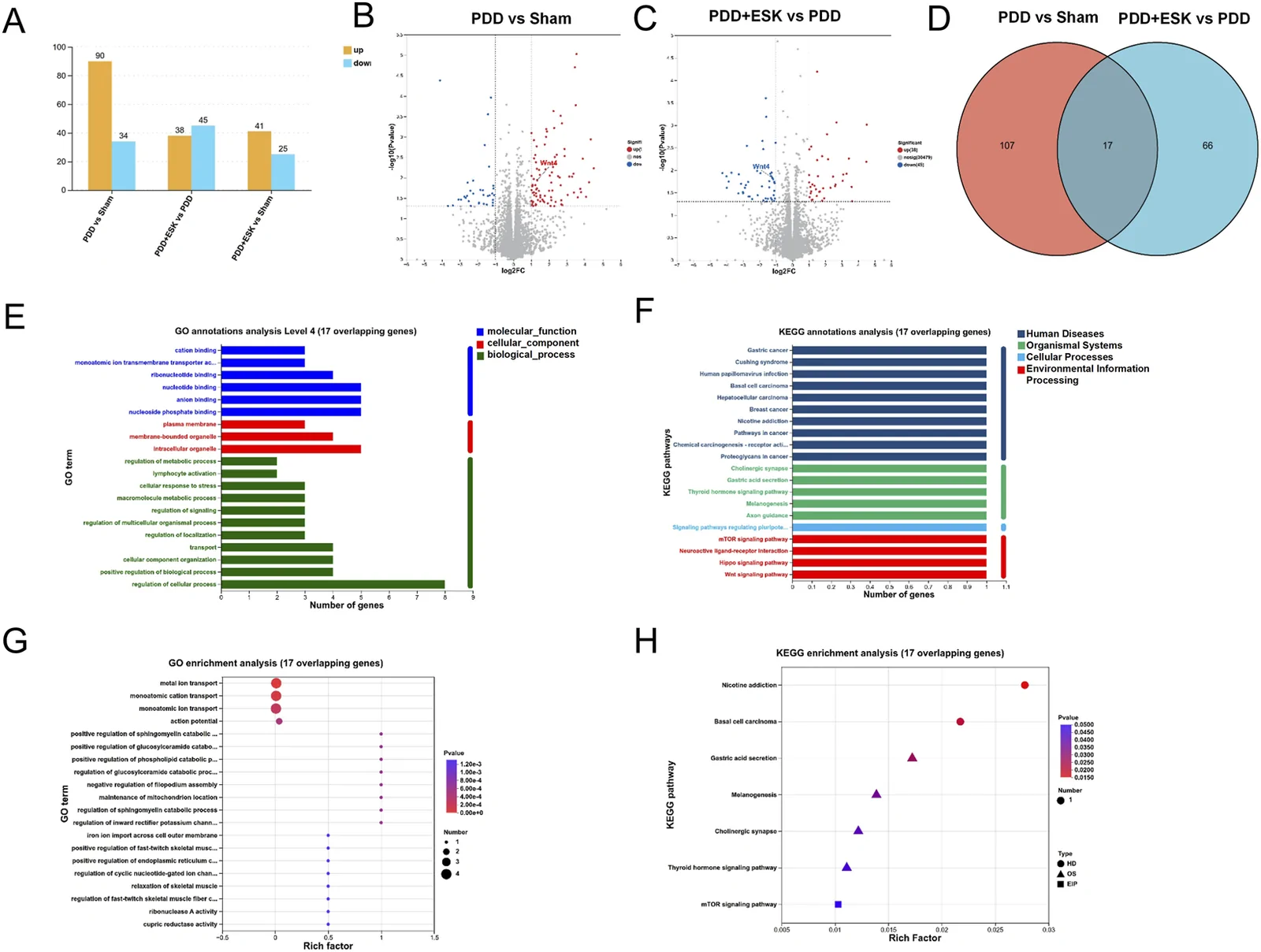
Exploratory Transcriptomic profiling and bioinformatic identification of overlapping esketamine-regulated candidate genes in the ACC. **(A)** Statistical quantification showing the absolute counts of upregulated (orange bars) and downregulated (light blue bars) candidate DEGs across different cohort comparisons, highlighting the overall transcriptomic alterations within the ACC. **(B,C)** Volcano plots illustrating the distribution of identified candidate transcripts for the PDD model group vs. Sham group comparison **(B)** and the PDD + ESK group vs. PDD group comparison **(C)**. Each point represents an individual gene; upregulated and downregulated genes are highlighted in red and blue, respectively (non-significant genes in gray). Nominal screening criteria were set at ∣log_2_ Fold Change∣≥ 1.0 and an unadjusted *P*-value <0.05.Candidate genes such as *Wnt4* are annotated. **(D)** Venn diagram displaying the intersection of candidate differentially expressed genes (DEGs) between the PDD vs. Sham and PDD + ESK vs. PDD comparisons (n = 5 independent biological samples per group), identifying 17 overlapping candidate genes prioritized for further investigation. **(E,F)** GO functional annotation **(E)** and KEGG functional classification **(F)** sorting the 17 overlapping candidate genes into biological categories. **(G,H)** Dot plots of GO enrichment analysis **(G)** and KEGG pathway enrichment analysis **(H)** showcasing prominently enriched terms and functional pathways (including the mTOR signaling pathway) for the 17 candidate genes. Color gradients represent statistical significance based on exploratory unadjusted *P*-value (*P* < 0.05).

#### 3.3.2 Exploratory functional enrichment and PPI network analyses

Gene Ontology (GO) and KEGG functional analyses were performed to characterize the exploratory biological profiles of candidate DEGs in the ACC (Supplementary Figures S2A–S5H). GO enrichment revealed that candidate DEGs in the PDD group were associated with functions such as potassium channel activity and ion transmembrane transport, whereas ESK intervention showed enrichment in processes related to dephosphorylation regulation, restoration of ion homeostasis, and synaptic remodeling (Supplementary Figures S2A–D). KEGG analysis indicated that candidate DEGs in PDD model rats were enriched in neuroactive ligand–receptor interactions, calcium signaling, and cell adhesion molecules. Following ESK treatment, candidate DEGs showed enrichment in pathways including PI3K-Akt signaling and extracellular matrix (ECM)-receptor interactions (Supplementary Figures S2E–S5H).

Protein-protein interaction (PPI) networks were constructed to evaluate potential candidate indicators (Supplementary Figures S2I,J). In the PDD *versus* Sham network, *Wnt4* emerged as an exploratory candidate node with a connectivity degree of 5, directly interacting with multiple genes such as *Wnt2b* and *Fgf8* (Supplementary Figure S2I). Following ESK intervention, *Wnt4* expression shifted from upregulation to downregulation, and its interaction profile was simplified, retaining connections with a few genes, including *Fgf10* (Supplementary Figure S2J). These exploratory bioinformatic findings suggest that *Wnt4* represents an exploratory observational candidate associated with ESK treatment, prioritized for subsequent orthogonal validation at the protein level.

#### 3.3.3 Overlapping candidate transcripts and pathway profiling

Through Venn diagram analysis of candidate DEGs identified from the PDD *versus* Sham and PDD + ESK *versus* PDD comparisons, a total of 17 overlapping genes were identified (Figure 4D; Table 1). These genes exhibited a reciprocal expression pattern between the two comparisons, such that genes upregulated (or downregulated) in the PDD group showed significant attenuation of expression following ESK intervention. This finding indicates that modulation of expression of these 17 genes is potentially relevant to the therapeutic effects of ESK, representing an exploratory transcriptomic signature underlying the pharmacological actions of ESK in mitigating PDD pathology.

GO annotation and enrichment analyses revealed that these 17 overlapping candidate genes were predominantly localized to the plasma membrane and membrane-associated structures (Figure 4E). Functional profiling indicated that these genes are primarily involved in transmembrane transport, metabolic regulation, and maintenance of action potentials (Figures 4E,G), which are essential for regulating neuronal excitability and synaptic homeostasis.

KEGG pathway analysis was subsequently performed to explore the metabolic and signaling networks of these candidate genes (Figure 4F). Under an exploratory threshold (unadjusted *P* < 0.05), these 17 overlapping candidate transcripts mapped to seven enriched functional pathways, including the cholinergic synapse and mTOR signaling pathways (Figure 4H; Table 2; Supplementary Table S3). Notably, the candidate transcript *Wnt4* was enriched in four of these pathways, highlighting its potential as an exploratory candidate node within the dysregulated network in PDD.

**TABLE 2:** KEGG pathways enriched from the 17 esketamine-regulated candidate genes.

Pathway ID	Pathway description	*P*-value	*P*adjust	First category	Associated gene
rno05033	Nicotine addiction	0.0181	0.3990	Human diseases	*Chrna4*
rno05217	Basal cell carcinoma	0.0231	0.2545	Human diseases	*Wnt4*
rno04971	Gastric acid secretion	0.0291	0.2135	Organismal systems	*Kcne2*
rno04916	Melanogenesis	0.0361	0.1983	Organismal systems	*Wnt4*
rno04725	Cholinergic synapse	0.0410	0.1803	Organismal systems	*Chrna4*
rno04919	Thyroid hormone signaling pathway	0.0449	0.1647	Organismal systems	*Wnt4*
rno04150	mTOR signaling pathway	0.0484	0.1520	Environmental information processing	*Wnt4*

KEGG, Kyoto Encyclopedia of Genes and Genomes. Pathway enrichment analysis was performed based on the 17 candidate gene set using the KEGG database (v2023.09) with the R*attus norvegicus* genome as the reference background. Unadjusted *P*-values and Benjamini-Hochberg FDR-adjusted *P*-values (*P*_adjust_) were calculated via hypergeometric testing. Enrichment filtering with an unadjusted *P* < 0.05 baseline was applied strictly as an exploratory screening strategy to identify candidate leads (e.g., *Wnt4*), with selected downstream targets subsequently validated through targeted protein-level assays (Western blotting for p-mTOR/p-GSK3β).

### 3.4 Orthogonal validation of esketamine-associated candidate markers within the ACC

To provide a preliminary validation of the transcriptomic screening, RT-qPCR analysis was conducted to evaluate the mRNA expression levels of candidate genes (including *Wnt4, Chrna4, Prkcd, and Ntng1*) within the ACC. The analysis revealed significant upregulation of these candidate genes in the PDD model group compared to the Sham group, which was attenuated following esketamine intervention (Figures 5A–D), showing concordance with the exploratory transcriptomic profiles.

**FIGURE 5:**
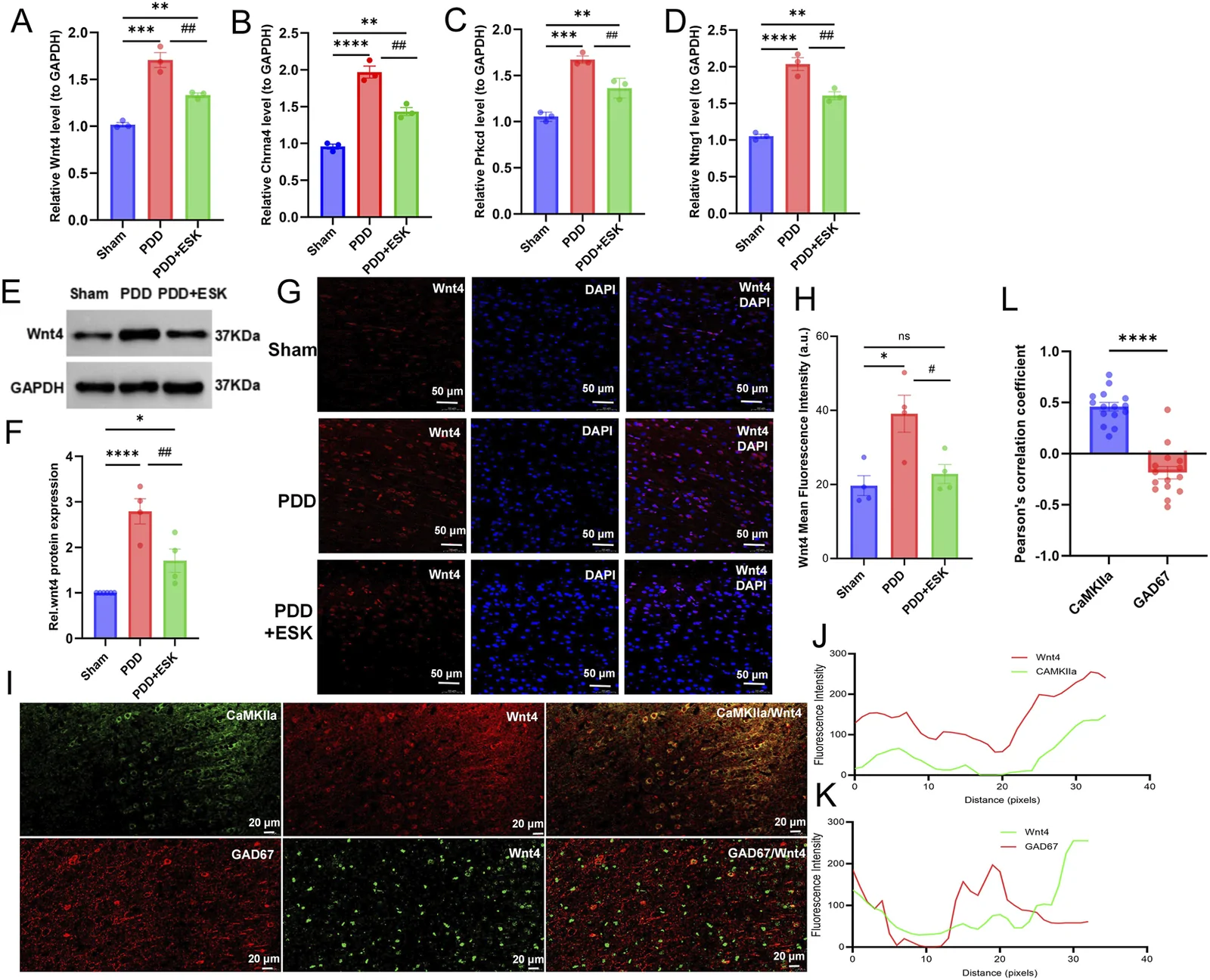
Transcriptomic validation, exploratory protein expression profiles, and cell-type-specific localization of screening-based candidate genes within the ACC. **(A–D)** RT-qPCR validation of candidate genes within the ACC, including **(A)** *Wnt4*, **(B)** *Chrna4,* **(C)** *Prkcd,* and **(D)** *Ntng1*, with mRNA expression levels normalized to *GAPDH*. Pairwise comparisons *via* one-way ANOVA followed by Tukey’s *post hoc* test revealed a significant transcriptional elevation in the PDD group (***P = 0.0001 for *Wnt4*; ****P < 0.0001 for *Chrna4*; ***P = 0.0002 for *Prkcd*; ****P < 0.0001 for *Ntng1* vs. Sham group), which was significantly attenuated following esketamine treatment (^##^ *P* = 0.0039 for *Wnt4*; ^##^ *P* = 0.0019 for Chrna4; ^##^ *P* = 0.0070 for *Prkcd*; ^##^ *P* = 0.0064 for *Ntng1* vs. PDD group). **(E,F)** Representative Western blot bands **(E)** and statistical quantification **(F)** of translational Wnt4 protein expression levels in the ACC homogenates, normalized to GAPDH (37 kDa); ****P < 0.0001 vs. Sham;^##^ *P* = 0.0067 vs. PDD. **(G,H)** Representative immunofluorescence micrographs **(G)** and quantitative analysis of Mean Fluorescence Intensity (MFI) **(H)** of Wnt4 immunoreactivity (red) counterstained with DAPI (blue) in the ACC (scale bars = 50 μm; *P = 0.0102 vs. Sham; ^#^ *P* = 0.0265 vs. PDD). **(I)** Double-immunofluorescence staining evaluating the cellular localization of Wnt4 within the ACC (scale bars = 20 μm). Top panels: Wnt4 (red) co-labeled with CaMKIIα (green, glutamatergic marker). Bottom panels: Wnt4 (green) co-labeled with GAD67 (red, GABAergic marker). **(J,K)** Fluorescence intensity line profiling curves tracking the spatial distribution and co-localization metrics of Wnt4 with CaMKIIα^+^ **(J)** and GAD67^+^ **(K)**. **(L)** Quantitative co-localization analysis comparing Pearson’s correlation coefficient (above threshold) of Wnt4 with CaMKIIα^+^ *versus* GAD67^+^ (****P < 0.0001). Data are presented as Mean ± SEM. Multiple comparisons (**A-D,F,H**) were evaluated *via* one-way ANOVA followed by Tukey’s *post hoc* test, and pairwise comparison (**L**) was analyzed *via* two-tailed unpaired Student’s t-test. Sample sizes: n = 3 for qPCR assays **(A–D)**; n = 4 for biochemistry and histology assays **(F,H)**; n = 15 individual neurons from N = 3 biological replicates per group for co-localization assay **(L)**. *P < 0.05, **P < 0.01, ***P < 0.001, ****P < 0.0001 vs. Sham group; ^#^ *P* < 0.05, ^##^ *P* < 0.01 vs. PDD group; ns, not significant.

Based on the collective bioinformatic screening filters-specifically the prominent counter-regulation profile across disease and treatment states, coupled with its active topological involvement across multiple enriched pathways including the mTOR signaling pathway-the transcript *Wnt4* was prioritized as an exploratory candidate marker for translational protein-level validation.

Further validation revealed a marked increase in Wnt4 protein expression in the ACC of the PDD group, as demonstrated by Western blot (Figures 5E,F) and immunofluorescence staining (Figures 5G,H). Esketamine treatment concurrently attenuated both its mRNA and protein levels, providing a preliminary validation of *Wnt4* expression profiles under esketamine regulation. To further explore its cellular distribution, double-immunofluorescence staining and representative intensity line profiling demonstrated partial signal overlap between Wnt4 and CaMKIIα-positive glutamatergic neurons, as well as GAD67-positive GABAergic interneurons (Figures 5I–K). Quantitative colocalization analysis revealed that Wnt4 exhibited a strong positive spatial correlation with CaMKIIα^+^ neurons but minimal correlation with GAD67^+^ interneurons (Figure 5L, Pearson’s r, ****P < 0.0001), indicating its preferential localization in excitatory neurons within the ACC.

### 3.5 Concomitant alterations in Wnt4-GSK3β-mTOR signaling cascades and synaptic protein profiles within the ACC under esketamine regulation

To explore the molecular microenvironment downstream of transcriptomic screening, we evaluated the protein profiles of the Wnt4–GSK3β–mTOR signaling cascade and synaptic plasticity-associated markers within the ACC. Western blot analysis revealed that, compared with the Sham group, the activation levels of downstream elements were altered in the ACC of PDD rats, characterized by significant elevations in the phosphorylation of GSK3β (p-GSK3β), mTOR (p-mTOR), and 4EBP1 (p-4EBP1). Conversely, the total protein abundances of GSK3β, mTOR, and 4EBP1 remained statistically unaltered across all experimental cohorts. Notably, ESK treatment effectively attenuated these concomitant signaling shifts, resulting in a downregulation of the phosphorylated forms of these cascade components (Figures 6A–I).

**FIGURE 6:**
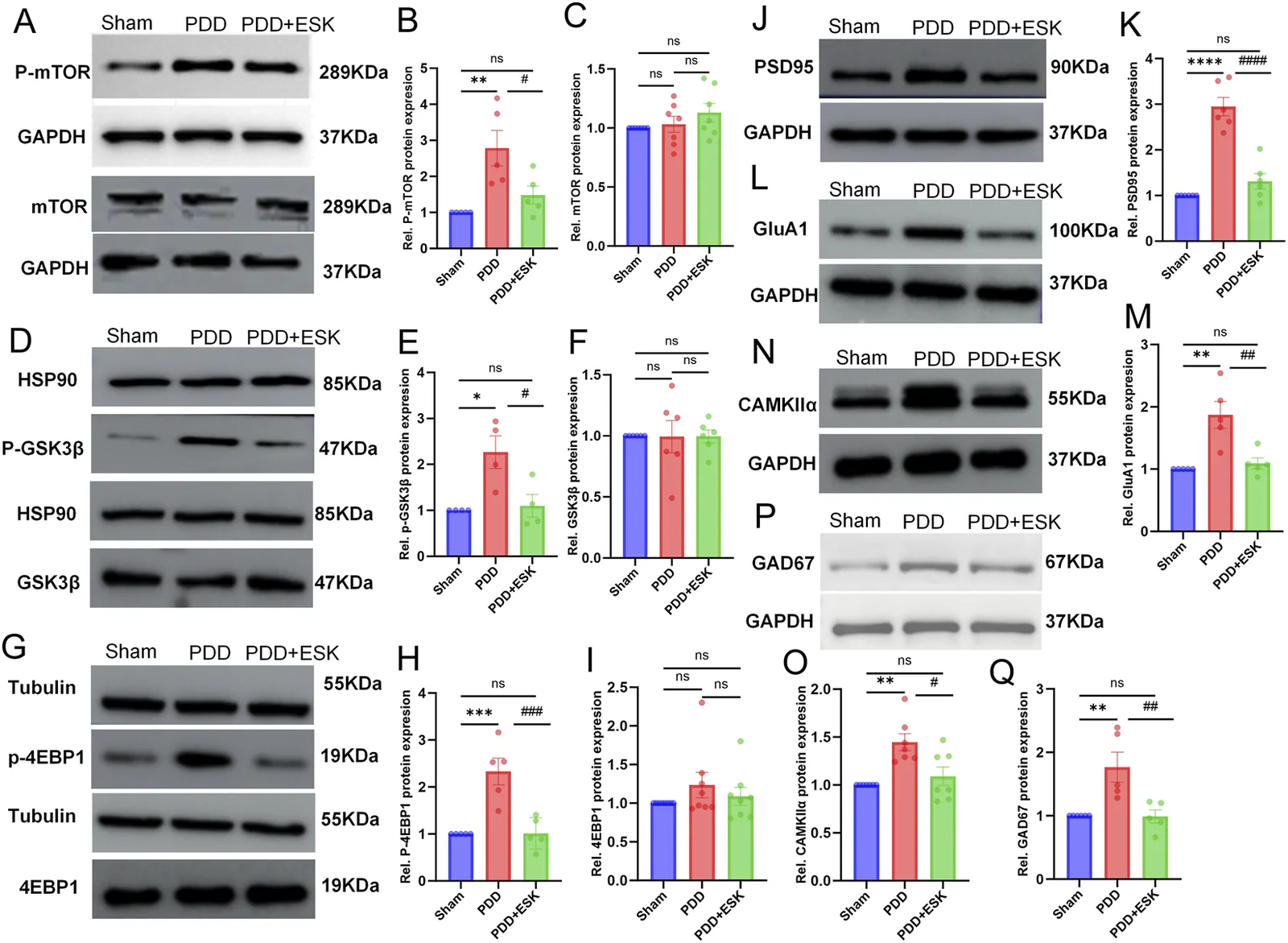
Western blot analysis of signaling pathway proteins and synaptic proteins within the ACC. **(A–I)** Representative Western blot bands and statistical quantification of relative protein expression levels for the mTOR/GSK3β/4EBP1 signaling pathway in ACC homogenates. Both phosphorylated and total levels of mTOR, GSK3β, and 4EBP1 were relative to their corresponding loading controls (GAPDH or HSP90). Pairwise comparisons *via* one-way ANOVA followed by Tukey’s *post hoc* test revealed a significant elevation of all phosphorylated isoforms in the PDD group (**B**: **P = 0.0049; E: *P = 0.0149; **(H)** ***P = 0.0008 vs. Sham group), which was significantly attenuated by esketamine intervention (**B**: ^#^ *P* = 0.0330; **(E)** ^#^ *P* = 0.0227; **(H)** ^###^ *P* = 0.0008 vs. PDD group). Total expression levels of mTOR, GSK3β, and 4EBP1 remained statistically unaltered across all groups (*P* > 0.05, ns), confirming that signaling shifts reflect altered phosphorylation states under invariant total kinase levels. Data are expressed as mean ± SEM. **(J–Q)** Representative Western blot bands and densitometric quantification of relative protein expression levels for critical synaptic and neuronal markers in ACC homogenates, including PSD95 **(J,K)**, GluA1 **(L,M)**, CaMKIIα **(N, O)**, and GAD67 **(P,Q)**. One-way ANOVA followed by Tukey’s *post hoc* test confirmed that all evaluated proteins were markedly upregulated in the PDD group (****P < 0.0001 for PSD95; **P = 0.0016 for GluA1 and CaMKIIα; **P = 0.0045 for GAD67 vs. Sham group) and significantly suppressed following esketamine treatment (^####^ *P* < 0.0001 for PSD95; ^##^ *P* = 0.0037 for GluA1; ^#^ *P* = 0.0104 for CaMKIIα; ^##^ *P* = 0.0055 for GAD67 vs. PDD group). Data are presented as Mean ± SEM. The exact biological sample sizes for each figure panel are as follows: n = 7 for **(C,O)**; n = 6 for **(F,K)**; n = 5 for **(B,E,H,Q)**; and n = 4 for **(I,M)**. Multiple comparisons were evaluated *via* ordinary one-way ANOVA followed by Tukey’s *post hoc* test. *P < 0.05, **P < 0.01, ***P < 0.001, ****P < 0.0001 vs. Sham group; ^#^ *P* < 0.05, ^##^ *P* < 0.01, ^###^ *P* < 0.001, ^####^ *P* < 0.0001 vs. PDD group; ns, not significant.

Assessment of synaptic proteins demonstrated significant increases in PSD95 and GluA1 expression in PDD rats, which were normalized following ESK intervention. In parallel, the excitatory neuronal marker CaMKIIα was significantly elevated in the PDD group, while the inhibitory neuronal marker GAD67 exhibited a trend toward increased expression; both alterations were partially attenuated by ESK (Figures 6J–Q).These findings indicate a robust molecular correlation, suggesting that ESK administration is closely associated with the concurrent stabilization of Wnt4-GSK3β-mTOR signaling cascades and the modulation of local excitatory/inhibitory synaptic protein abundance homeostasis within the ACC, rather than establishing a definitive causal mediation or functional synaptic circuit current restoration.

## 4 Discussion

### 4.1 Exploratory transcriptomic profiling and orthogonal molecular validation identify Wnt4 as a candidate signaling component in PDD-associated ACC dysfunction

NP is frequently accompanied by depressive symptoms, posing significant challenges for effective treatment, and although ESK has shown promise in alleviating both conditions, its central mechanisms remain incompletely understood. The ACC, an anatomical structure highly involved in integrating nociceptive and affective information, has been widely documented to participate in both chronic pain and depression (Journée et al., 2023; Barthas et al., 2015; Li et al., 2025). In the present study, our findings suggest that the ACC serves as an important neural substrate associated with PDD, as evidenced by markedly increased c-Fos expression compared with other brain regions. This hyperactivation was predominantly localized to CaMKIIα-positive excitatory neurons, accompanied by elevated glutamate levels and enhanced neuronal calcium responses to noxious stimulation, which implies a state of altered neural activity and synchronized cellular fluctuations within cortical circuitries (Wang et al., 2024; Gao et al., 2017). Intriguingly, our ultrastructural analysis revealed reduced synaptic density, supporting the recent concept that such aberrant functional hyperactivation within the ACC could be concurrent with pathological synaptic deficiency and neural circuit remodeling (Wang et al., 2025; Kessels et al., 2025; Yang et al., 2015).

Notably, ESK treatment mitigated these abnormalities, supporting its role in modulating neuronal and synaptic homeostasis under PDD conditions. To further elucidate the molecular alterations associated with these synaptic phenotypes, we performed transcriptomic profiling of the ACC tissue. While whole-transcriptome multiple-testing correction did not yield statistically significant differences after FDR adjustment, nominal exploratory screening (∣log_2_ FC∣≥1.0, unadjusted *P* < 0.05) allowed us to generate data-driven biological hypotheses and prioritize candidate transcripts. Comparative analysis revealed a discrete subset of transcripts that were dysregulated in the PDD state and subsequently mitigated following ESK intervention. To orthogonally validate these exploratory bioinformatic leads, selected candidate genes were evaluated by RT-qPCR and translational protein-level assays. Crucially, our qRT-PCR results demonstrated that the mRNA expression of *Wnt4* was significantly upregulated in PDD rats and effectively attenuated by ESK treatment, exhibiting a directional alteration highly consistent with the high-throughput sequencing dataset.

Furthermore, this validated expression pattern was accompanied by synchronous transcriptional fluctuations of other co-regulated genes, including *Chrna4* (previously identified in clinical cohorts as a genetic susceptibility locus linked to depressive phenotypes and treatment responses (Yu et al., 2019; Csala et al., 2017)), *Prkcd* (a characterized regulator of spine-specific stability and synaptic plasticity (Colgan et al., 2023)), and *Ntng1* (involved in localized axonal guidance and emotional circuit formation (Zhang et al., 2016)), all of which were similarly verified by qRT-PCR. Beyond these canonical synaptic elements, functional pathway annotations for non-neuronal overlapping DEGs-specifically *Steap4, Col26a1,* and *Eddm3b*-suggest the involvement of non-cell-autonomous mechanisms in ACC dysfunction. Specifically, *Steap4* (a metalloreductase modulating iron homeostasis and oxidative stress) and *Col26a1* (an extracellular matrix component) implicate microglial/astrocytic inflammatory responses and neurovascular matrix remodeling, both of which dynamically modulate neuronal excitability and synaptic structural plasticity. Additionally, *Eddm3b* represents a transcript candidate involved in neurosecretory microenvironmental adaptations. Collectively, the dysregulation and subsequent modulation of these molecular targets indicate that microenvironmental perturbation accompanies neuronal synaptic pathology in PDD, providing a complementary molecular framework for characterizing downstream synaptic receptor plasticity and signaling cascades.

### 4.2 Exploratory transcriptomic profiles and intracellular cascades in ACC dysfunction under PDD

The overlapping dataset of 17 candidate genes co-corrected by esketamine (ESK) provides a molecular template suggesting that PDD stems from a coordinated, multi-level network disturbance rather than isolated molecular lesions. Within this framework, GO and KEGG pathway analyses mapped significant enrichment onto neuroplasticity, cholinergic synapses, and mTOR pathways, implicating *Wnt4* as a potential upstream regulatory node linking extracellular cues to downstream metabolic and structural adaptations.

Within the neuroplasticity and transmission clusters, transcriptomic data screened out a synchronized disturbance in cortical microarchitecture and neurotransmitter signaling. On one hand, canonical Wnt signaling modulates glycogen synthase kinase-3 beta (GSK3β) to preserve dendritic microarchitecture, and its regional aberrations correlate with mechanical hypersensitivity and affective despair (Ramos-Fernández et al., 2019; Wang et al., 2019). On the other hand, transcriptomic annotation revealed a profound disturbance in neuroactive ligand-receptor interactions, a signature implying a generalized homeostatic and signaling imbalance within the central nervous system during comorbidity development (Xia et al., 2026). Within this altered network architecture, intersecting the DEG datasets identified the cholinergic nicotinic receptor subunit Chrna4 as an aberrant node, which exhibited a marked pathological upregulation in the PDD state and subsequent downregulation following ESK treatment. This transcription-level fluctuation indicates that aberrant cholinergic transmission within the ACC framework potentially contributes to the concurrent development of mechanical hypersensitivity and comorbid affective deficits.

Network topology positions the intracellular mTOR cascade as a prominent downstream signaling cascade intersecting with these upstream inputs *via* progressive GSK3β phosphorylation (Aref et al., 2025; Kim and Lee, 2026). This bioinformatic mapping aligns with established empirical evidence showing that anti-allodynic effects are mediated by suppressing the pathological hyperactivation of the mTOR pathway specifically within the ACC to mitigate structural impairments in synaptic scaffolding (Wehle et al., 2026). Collectively, these findings indicate that PDD involves a coordinated disruption of a Wnt4-GSK3β-mTOR intersection within the ACC, offering a data-driven exploratory rationale to independently evaluate this pathway at the protein and phosphorylation levels.

### 4.3 Esketamine modulation of a Wnt4-centered candidate network module within the ACC

Topological decomposition of the aggregate PPI network further suggested that ESK exposure correlates with the architectural preservation of a discrete functional sub-network cluster structured around Wnt4 (Qin et al., 2022; Zhou et al., 2018; Aguilar-Valles et al., 2021). This distinct molecular module structurally encompasses elements associated with synaptic organization, localized axonal pathfinding guidance, and neuroimmune regulatory factors. Based on these bioinformatic predictions and integrating KEGG pathway architecture with neurobiological evidence, we examined how these network elements are postulated to converge onto the central Wnt4-GSK3β-mTOR axis to associate with altered cortical synaptic plasticity during the pathological PDD state.

Within this interaction framework, *Wnt4* serves as a prominent candidate node and an inferred upstream modulator of the intracortical GSK3β-mTOR cascades, whose aberrant expression correlates with atypical synaptic remodeling and microarchitectural disruption within localized cortical circuitries (Kim and Monteggia, 2020; McLeod et al., 2018). While our functional evaluation focuses on this primary cascade, the prominent topological positioning of this locus within the transcriptomic dataset aligns with clinical findings identifying Wnt signaling components within the pathophysiology of major depressive disorder and related neuropsychiatric conditions (Sanchez-Ruiz et al., 2024). The structural integration of these distinct molecular candidates within the predicted network module conceptually suggests that PDD pathology involves a multi-axial disruption of cortical synaptic integrity-characterized by compromised Wnt-mediated structural maintenance and diminished synaptic matrix organization.

Notably, the nominal transcriptomic enrichment of the mTOR cascade (unadjusted *P* = 0.0484) primarily reflected the core contribution of Wnt4. Recognizing the single-gene nature of this initial hit, pathway-level involvement was prudently interpreted and independently substantiated at the protein signaling level *via* Western blotting (measuring Wnt4, p-GSK3β, and p-mTOR), thereby providing robust orthogonal empirical evidence for the engagement of the ACC Wnt4-mTOR-GSK3β signaling pathway. Rather than operating *via* a restricted downstream neuronal mechanism, the pharmacological profile of ESK is hypothesized to involve broad-spectrum regulation of this upstream network architecture. By preserving the topological arrangement of this Wnt4-associated module, ESK concurrently dampens these multi-axial pathological disruptions, thereby facilitating the alleviation of local neural homeostasis and stabilizing downstream GSK3β-mTOR-dependent synaptic plasticity. This network-level regulation model provides a distinct framework for evaluating the multifaceted pharmacological actions of ESK.

### 4.4 Orthogonal validation of Wnt4-associated signaling markers and an exploratory mechanistic hypothesis

To orthogonally validate the exploratory candidate leads derived from our transcriptomic mapping, we quantified hub protein expression and phosphorylation activation within the rat ACC. Our Western blot analysis showed that Wnt4 protein expression is significantly upregulated in the ACC of PDD rats, with concomitant increases in p- GSK3β (Ser9), p-mTOR, and p-4EBP1, while total protein levels of GSK3β, mTOR, and 4EBP1 remained unaltered. Inhibitory phosphorylation of GSK3β at Ser9 reduces its kinase activity, thereby disinhibiting mTORC1 to facilitate downstream 4EBP1 phosphorylation and stimulate protein synthesis at synapses (Kavalali and Monteggia, 2025). Consistent with this signaling cascade, expression of the synaptic scaffolding markers PSD-95 and GluA1 was markedly elevated, suggesting that potential hyperactivation of intracortical mTOR signaling correlates with synaptic protein accumulation and atypical morphological plasticity (Tan et al., 2024). These protein-level findings provide translational alignment with our transcript-level prediction that localized Wnt4-associated signaling alterations co-occur with synaptic microarchitectural remodeling in the sensitized ACC.

Crucially, ESK treatment significantly attenuated these molecular alterations, including Wnt4 expression, downstream phosphorylation markers, and synaptic proteins. This therapeutic downregulation of the pathologically elevated p-mTOR cascade aligns with established empirical evidence showing that peripheral nerve injury drives a baseline hyperactivation of the mTOR pathway specifically within the ACC (Woo Um et al., 2019), and that pharmacological suppression of this hyperactive axis within the ACC simultaneously alleviates hypersensitivity and mitigates comorbid depressive-like behaviors (Liu et al., 2025). These convergent findings suggest that the therapeutic efficacy of ESK may extend beyond isolated receptor blockade to participate in the pharmacological modulation of these candidate upstream regulatory networks and dampen regional neuronal hyperactivity (Kowalczyk et al., 2025; Kim et al., 2023).

The divergent regulatory direction of mTOR signaling observed here reflects key differences in brain region, pathological baseline, and sampling kinetics compared with classical depression models. In traditional major depressive disorder, ketamine transiently activates mTORC1 in the medial prefrontal cortex (mPFC) and hippocampus (HPC) within 1–2 h to rescue compromised baseline protein synthesis (Aguilar-Valles et al., 2021). In contrast, in our chronic constriction injury-induced PDD model, persistent nociceptive input drives localized anterior cingulate cortex (ACC) hyperexcitability and sustained PI3K/Akt/mTOR pathway hyperactivation (Woo Um et al., 2019). Under such conditions, pharmacological inhibition, rather than activation, of ACC mTOR signaling alleviates both hypersensitivity and depressive-like behaviors (Liu et al., 2025). Furthermore, our delayed tissue evaluation (≥24–72 h post-administration) likely captures the sustained resolution of central sensitization rather than acute synaptogenic induction. Thus, the regulatory effect of ESK on mTOR signaling appears to be state- and circuit-dependent, potentially acting to attenuate pathologically elevated baseline activity within the sensitized ACC.

Simultaneously, we observed concurrent alterations in CaMKIIα and GAD67 protein levels, suggesting a potential perturbation in the baseline homeostatic ratio of complementary excitatory and inhibitory molecular markers (Luo et al., 2026). This biochemical shift is supported by our immunofluorescence data showing that c-Fos activity predominantly colocalizes with CaMKIIα-positive glutamatergic neurons, identifying them as the principal active cell population associated with the regional network hyperactivation typically observed in pain–depression comorbidity (Becker et al., 2023).

Based on these combined transcriptomic predictions, focused protein validations, and immunofluorescence mappings, we propose a plausible pharmacological mechanism hypothesis: Upregulated Wnt4 expression within the ACC-observed in the context of comorbid pain and depression-is accompanied by the activation of downstream GSK3β–mTOR signaling components, co-occurring with the upregulation of local synaptic markers (CaMKIIα and GAD67) and atypical synaptic ultrastructural remodeling, which, together with regional neuronal hyperexcitability and calcium hyperreactivity, ultimately track with the maintenance of the behavioral phenotypes of hyperalgesia and affective despair. Conversely, sub-anesthetic esketamine administration is closely accompanied by the concurrent modulation of these ACC Wnt4-associated signaling components, showing a restorative trend in synaptic ultrastructure and attenuated ACC hyperactivation, thereby sustaining its therapeutic anti-allodynic and antidepressant-like actions (Figure 7). This framework conceptualizes a novel facet of ESK pharmacology: while its rapid-onset effects are typically initiated *via* acute NMDA receptor antagonism, its sustained therapeutic efficacy appears to involve alternative modulations of upstream molecular cascades, functioning as a pharmacological modulator of cortical gene expression within the ACC.

**FIGURE 7:**
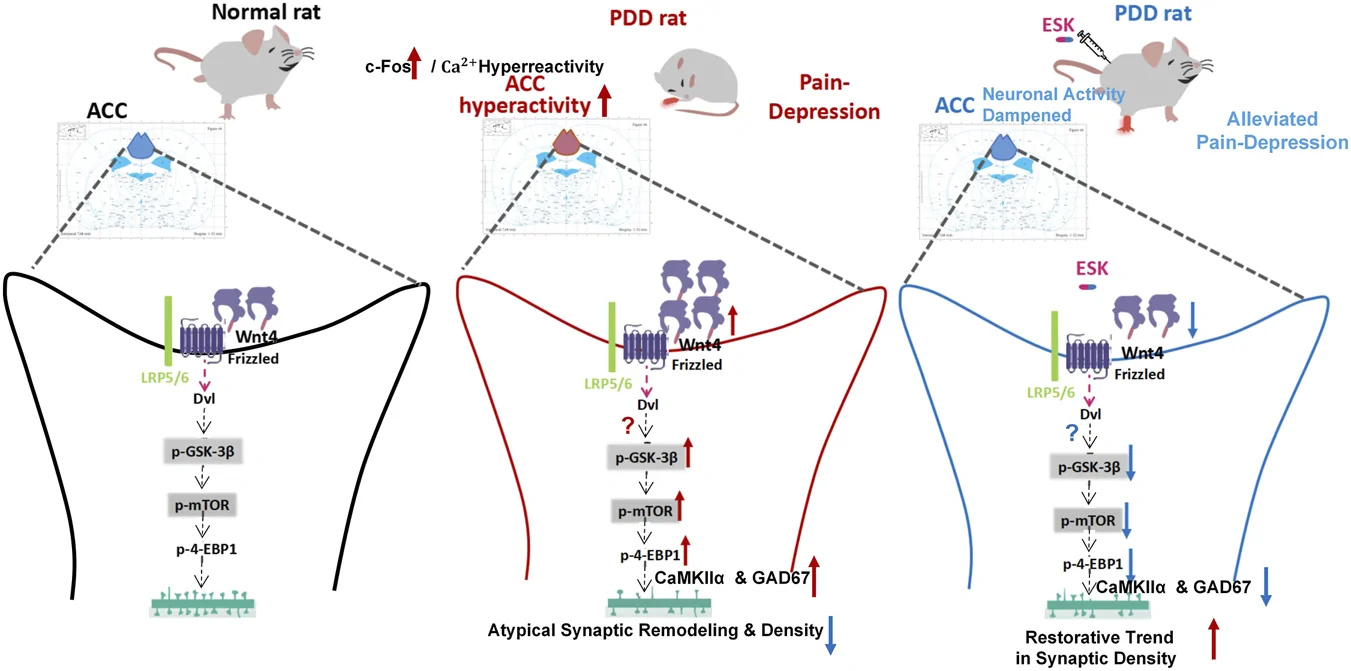
Proposed schematic model of inferred Wnt4-associated molecular cross-talk and synaptic adaptations in the ACC following sub-anesthetic esketamine administration. Note: Nodes enclosed in rectangles with shaded backgrounds (Wnt4, p-GSK-3β, p-mTOR, and p-4EBP1) represent candidate molecular markers directly evaluated *via* Western blotting in this study. The upstream membrane receptors and structural signaling intermediates (LRP5/6, Frizzled, and Dvl) and their connecting trajectories represent a proposed sequence inferred from KEGG/STRING databases and consensus literature. Dashed arrows accompanied by question marks (?) explicitly indicate inferred or proposed molecular cross-talk and parallel downstream adaptations rather than an experimentally validated linear cascade. In the PDD state, alterations among these candidate markers co-occur with the upregulation of synaptic-associated proteins (CaMKIIα and GAD67), atypical synaptic remodeling, and reduced synaptic density, alongside regional ACC neuronal hyperexcitability and calcium hyperreactivity (c-Fos↑ and Ca^2+^ hyperreactivity↑). Sub-anesthetic ESK administration is accompanied by the concurrent modulation of Wnt4-associated signaling components, a restorative trend in synaptic ultrastructure, and attenuated ACC hyperactivation, offering exploratory insights into its therapeutic anti-allodynic and antidepressant-like actions.

## 5 Conclusion

This study indicates that PDD is associated with ACC hyperactivity manifested within CaMKIIα-positive excitatory neurons, occurring alongside exploratory transcriptomic dysregulations featuring *Wnt4*. Our molecular and functional validations reveal that a single sub-anesthetic ESK dose effectively alleviates PDD behavioral phenotypes in rats. This therapeutic efficacy is associated with the modulation of the Wnt4-GSK3β-mTOR candidate signaling cascade, alongside the mitigation of downstream synaptic molecular deficits within ACC excitatory neurons. Collectively, this work provides supportive multi-level evidence for cortical molecular characterization in pain-related affective disorders and highlights a candidate signaling pathway for future therapeutic exploration.

## Data Availability

The data presented in the study are deposited in the GEO (Gene Expression Omnibus) repository, accession number GSE330695.
