# Supplementary material for: Esketamine alleviates the pain-depression dyad: insights from exploratory transcriptomic profiling and Wnt4-associated molecular alterations within the anterior cingulate cortex
Source: Front Pharmacol. 2026 Oct 16;17:1873924. doi: 10.3389/fphar.2026.1873924 (PMC13719547; doi:10.3389/fphar.2026.1873924)
Supplement: Supplementary file 1 [file DataSheet1.pdf]

Supplementary Table S1. Summary of experimental assays, sample sizes per group, and replicate specifications.

Assay	Sham(n)	PDD(n)	PDD+ESK(n)	ReplicateType
Behavioral Testing (CCI/OFT/FST)	6	6	6	Independent animals
RNA-seq	5	5	5	Independent animals
RT-qPCR	3	3	3	Independent animals
Glutamate ELISA	6	6	6	Independent animals
Immunofluorescence	4	4	4	Independent animals
Transmission Electron Microscopy	6	6	6	Independent animals
Fiber Photometry	5	5	5	Independent animals
HE Staining	3	3	3	Independent animals
Western Blotting	8	8	8	Independent animals

Abbreviations: CCI, chronic constriction injury; OFT, open field test; FST, forced swimming test; RNA-seq, RNA sequencing; RT-qPCR, reverse transcription-quantitative polymerase chain reaction; ELISA, enzyme-linked immunosorbent assay; HE, hematoxylin and eosin; PDD, pain-depression comorbidity; ESK, esketamine.

Note: The value in each group column represents the number of independent biological replicates (n, individual animals) utilized for each corresponding assay. All tissue samples were derived from distinct experimental animals to ensure independent biological validation.

Supplementary Table S2. Detailed primer sequences and parameter specifications used for RT-qPCR validation.

GeneName	AccessionNo.	Primersequence（5`-3`）		Tm (℃)	GC (%)	ProductLength （bp)
R-Gapdh	NM_017008.4	Sense	GCCAAGGTCATCCATGACAAC	59.8	52.4	152
		Antisense	GTGGATGCAGGGATGATGTTC	60.2	61.1	
R-Wnt4	NM_053402	Sense	TACGCCATCTCTTCAGCAGG	59.5	55	197
		Antisense	CCCTTGCTTCTCTCTCGGAC	59.8	60	
R-Chrna4	NM_024354	Sense	GCCATACGACTCGTCAGTCA	59.6	55	125
		Antisense	TCTCCAACCAGGCCCATAGT	60.3	55	
R-Prkcd	NM_133307	Sense	CCATAAGAAATGCATCGACAAG	57.1	40.9	130
		Antisense	CATGTAGTTATAGACCTTGAATCGG	57.1	40	
R-Ntng1	NM_001106465	Sense	TGTACAAGACAGCAGAGGGAG	59.1	52.4	139
		Antisense	CCTTGCCCACACATTTGTCAC	60.3	52.4	

Abbreviations: RT-qPCR, reverse transcription-quantitative polymerase chain reaction; Tm, melting temperature; GC, guanine-cytosine content; bp, base pair; NCBI, National Center for Biotechnology Information.

Note: The prefix "R-" explicitly denotes Rattus norvegicus (rat) target genes. All listed accession numbers refer to verified reference sequences deposited in the NCBI GenBank database
