# Supplementary material for: Esketamine alleviates the pain-depression dyad: insights from exploratory transcriptomic profiling and Wnt4-associated molecular alterations within the anterior cingulate cortex
Source: Front Pharmacol. 2026 Oct 16;17:1873924. doi: 10.3389/fphar.2026.1873924 (PMC13719547; doi:10.3389/fphar.2026.1873924)

Figure S1. Global transcriptomic expression heatmaps across experimental groups.

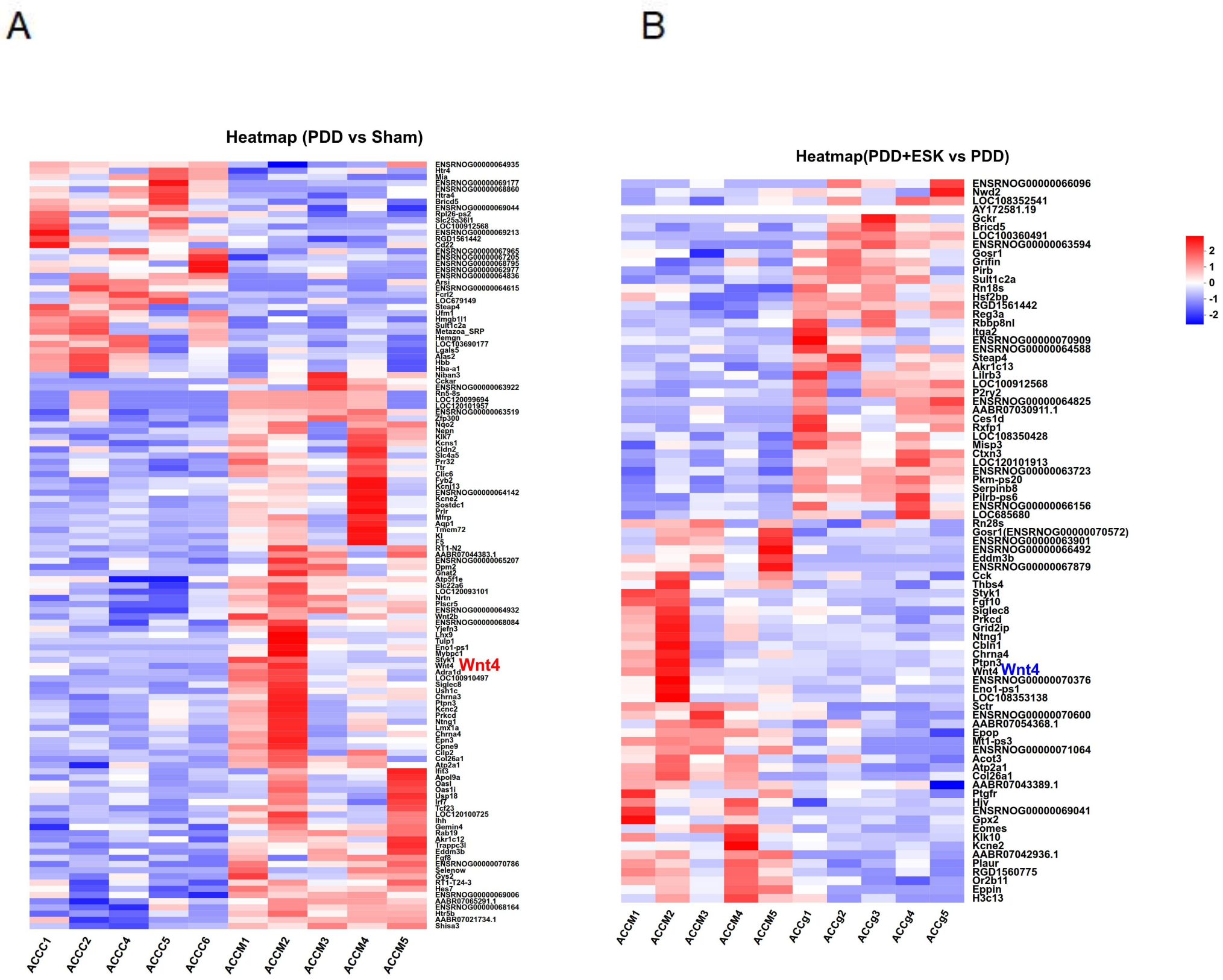

(A, B) Hierarchical clustering heatmaps mapping the expression profile patterns of prominent DEGs for the PDD vs. Sham group **(A)** and the PDD+ESK vs. PDD group **(B)**. The color gradient reflects the normalized gene expression level levels, shifting from relative upregulation (red) to downregulation (blue).

Figure S2. Functional enrichment and protein-protein interaction (PPI) network analyses of differentially expressed genes (DEGs) in the ACC

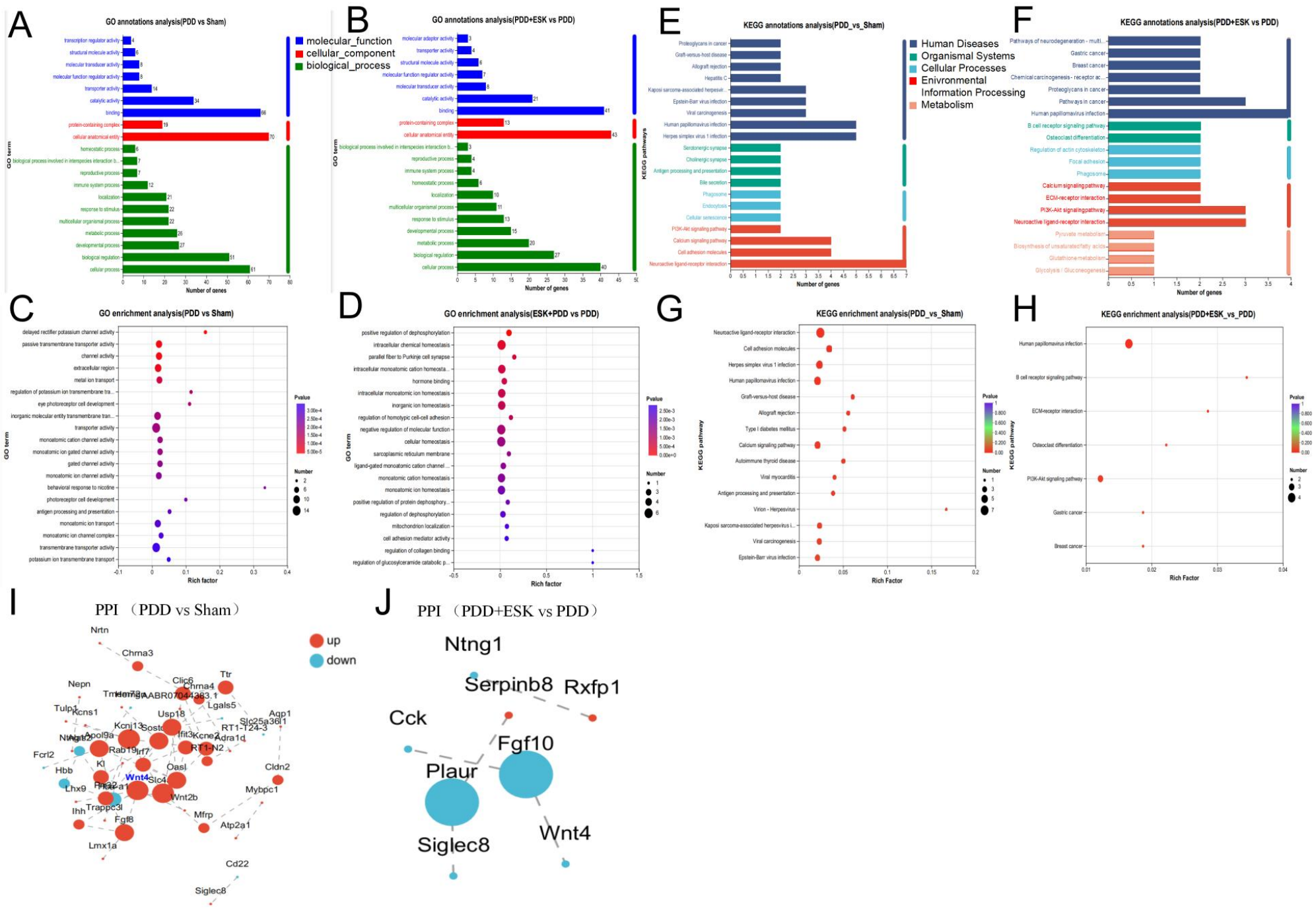

(A, B) GO functional annotation analysis classifying the identified DEGs into biological processes (BP), cellular components (CC), and molecular functions (MF) for the PDD model vs. Sham comparison (A) and the PDD+ESK vs. PDD comparison (B).

(C, D) Top enriched GO terms represented by dot plots for DEGs from the PDD model vs. Sham (C) and PDD+ESK vs. PDD groups (D) comparison.

(E, F) KEGG functional classification mapping DEGs to hierarchical biological categories for PDD vs. Sham (E) and the PDD+ESK vs. PDD (F).

(G, H) KEGG pathway enrichment dot plots showcasing the most prominently altered physiological pathways between PDD vs. Sham (G) and PDD+ESK vs. PDD (H) groups.

(I, J) PPI network of interconnected DEGs constructed via the STRING database for PDD vs. Sham (I) and PDD+ESK vs. PDD (J) comparisons, positioning *Wnt4* as a prominent topological candidate gene within the esketamine-responsive genomic cluster.

Bioinformatic Parameters and Criteria: DEGs were defined based on the thresholds of |log2 Fold Change| ≥1.0 and an unadjusted *P*-value < 0.05 utilizing the DESeq2 R package (n = 5 independent biological samples per group). GO and KEGG enrichment analyses were executed via goatools (v1.4.4) and the KEGG database (v2023.09), using the *Rattus norvegicus* (mRatBN7.2) genome as background. Statistical significance enrichment was set at an unadjusted *P*-value < 0.05. In panels (C, D, G, H), dot color indicates the *P*-value and dot size indicates the enriched gene count.PPI network (I, J) were generated via the STRING database (V12.0) on the Majorbio Cloud platform with disconnected nodes hidden. Node sizes is proportional to their topological degree of connectivity (representing statistical centrality); red and blue nodes denote upregulated and downregulated genes, respectively .

Supplementary Figure S3: Original Uncropped Western Blot Membranes

Target 1: c-fos (58 kDa) + Loading Control GAPDH (37 kDa)
(Corresponding to **Figure 2C**)

c-fos ~58 kDa

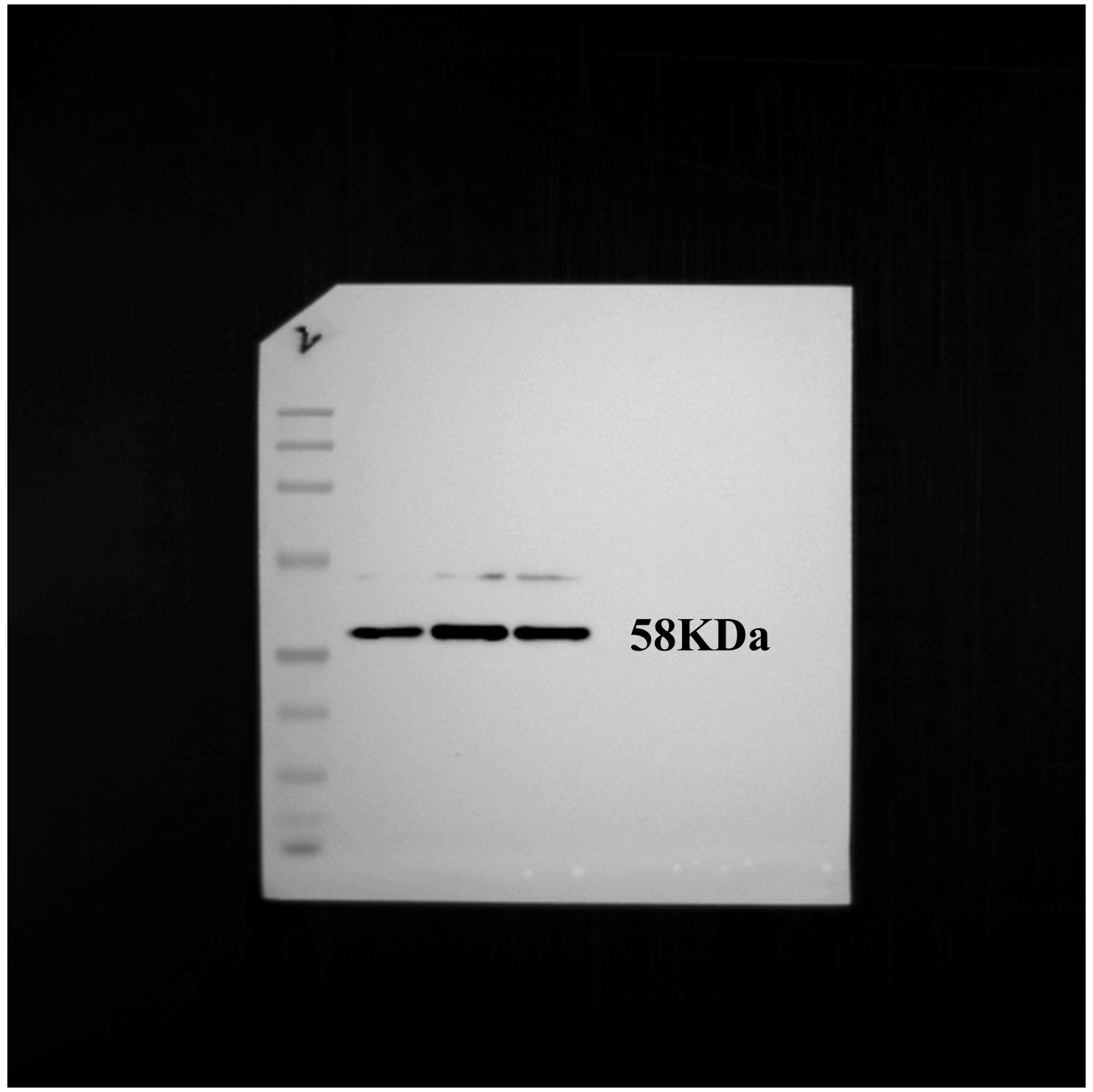

GAPDH ~37 kDa

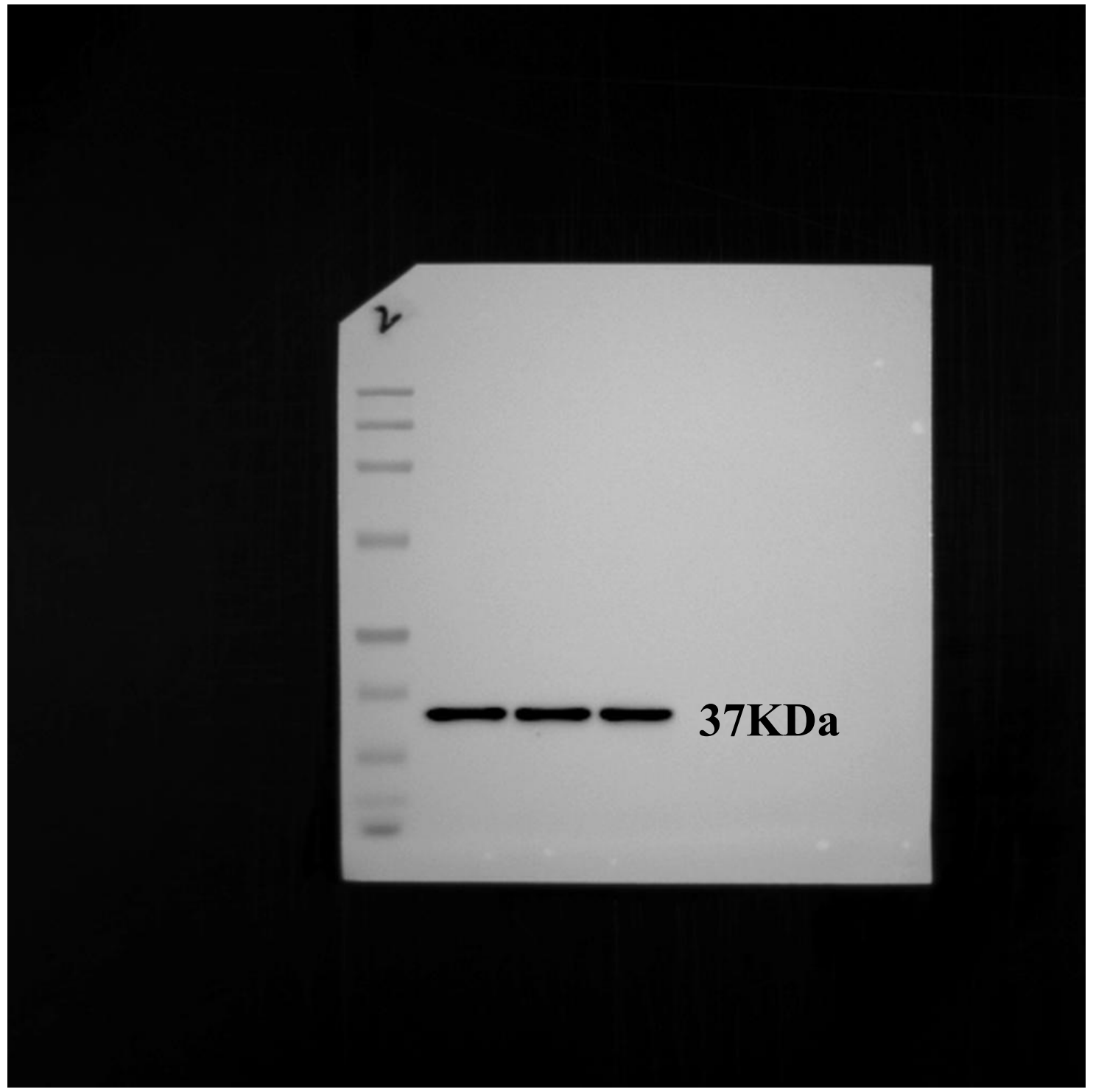

Target 2: Wnt4 (39 kDa) + Loading Control GAPDH (37 kDa)
(Corresponding to **Figure 5E**)

Wnt4 ~39 kDa

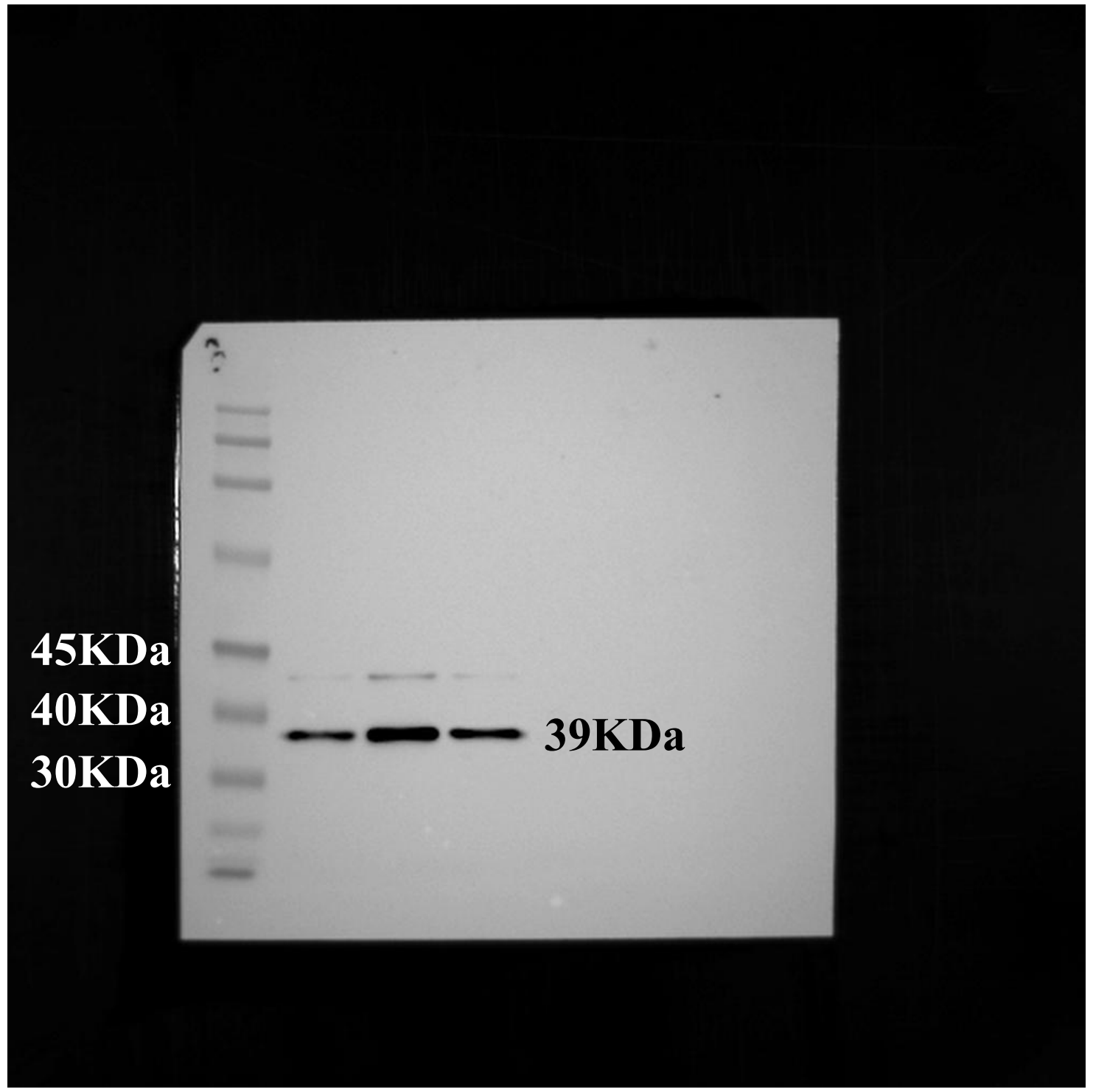

GAPDH ~37 kDa

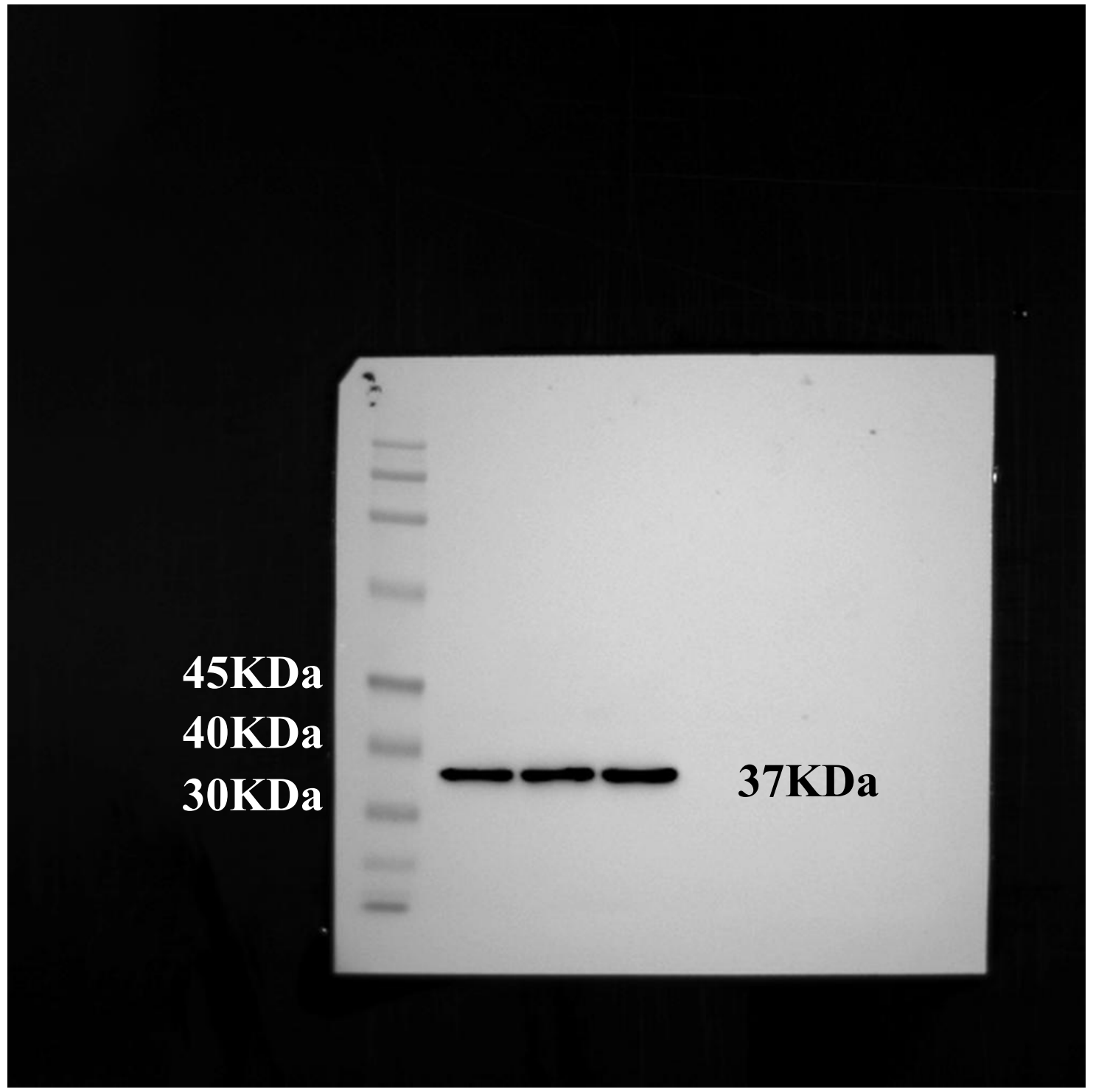

Target 3: p-mTOR (289 kDa) + Loading Control GAPDH (37 kDa)
(Corresponding to **Figure 6A**)

p-mTOR ~289 kDa

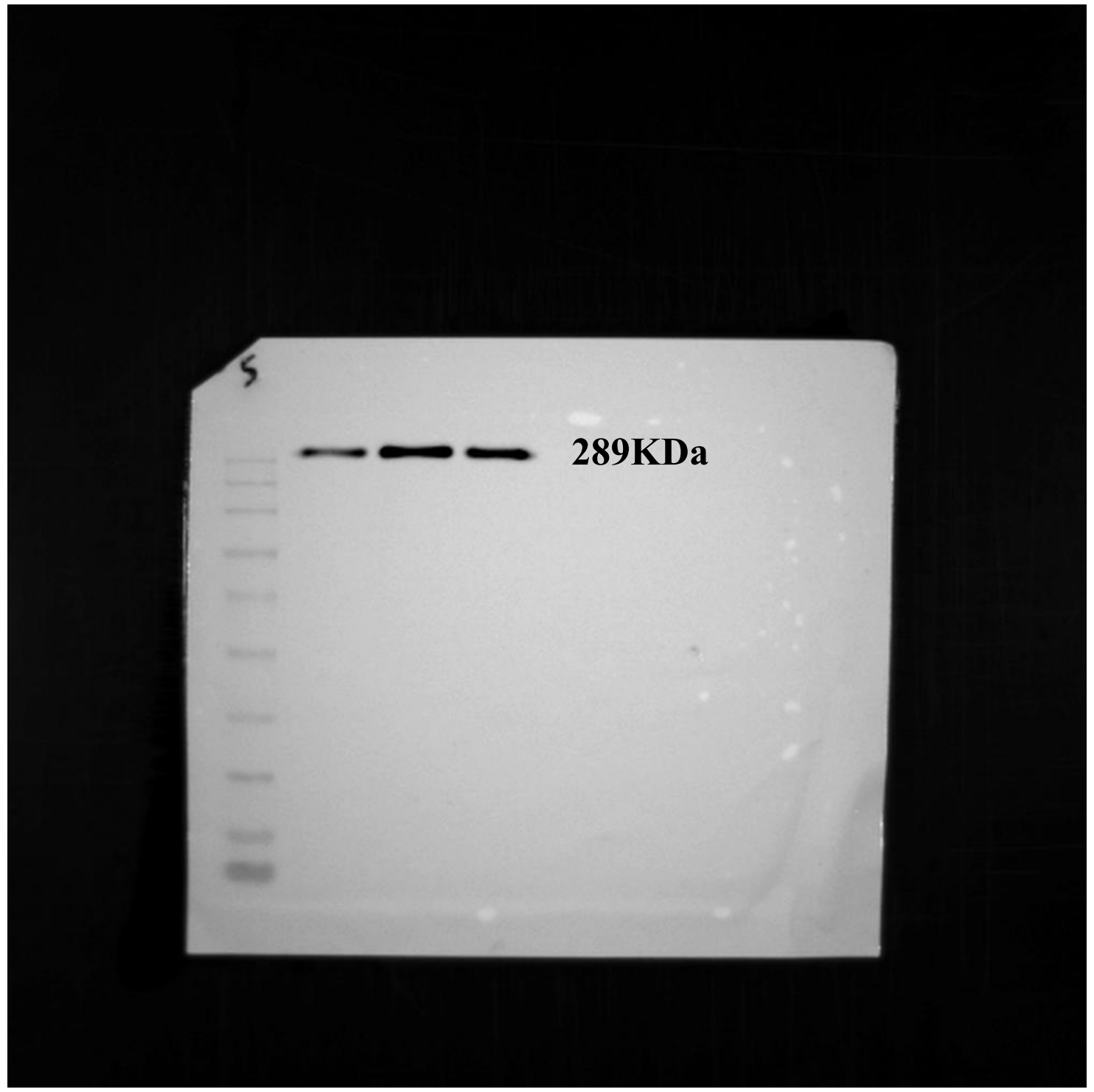

GAPDH ~37 kDa

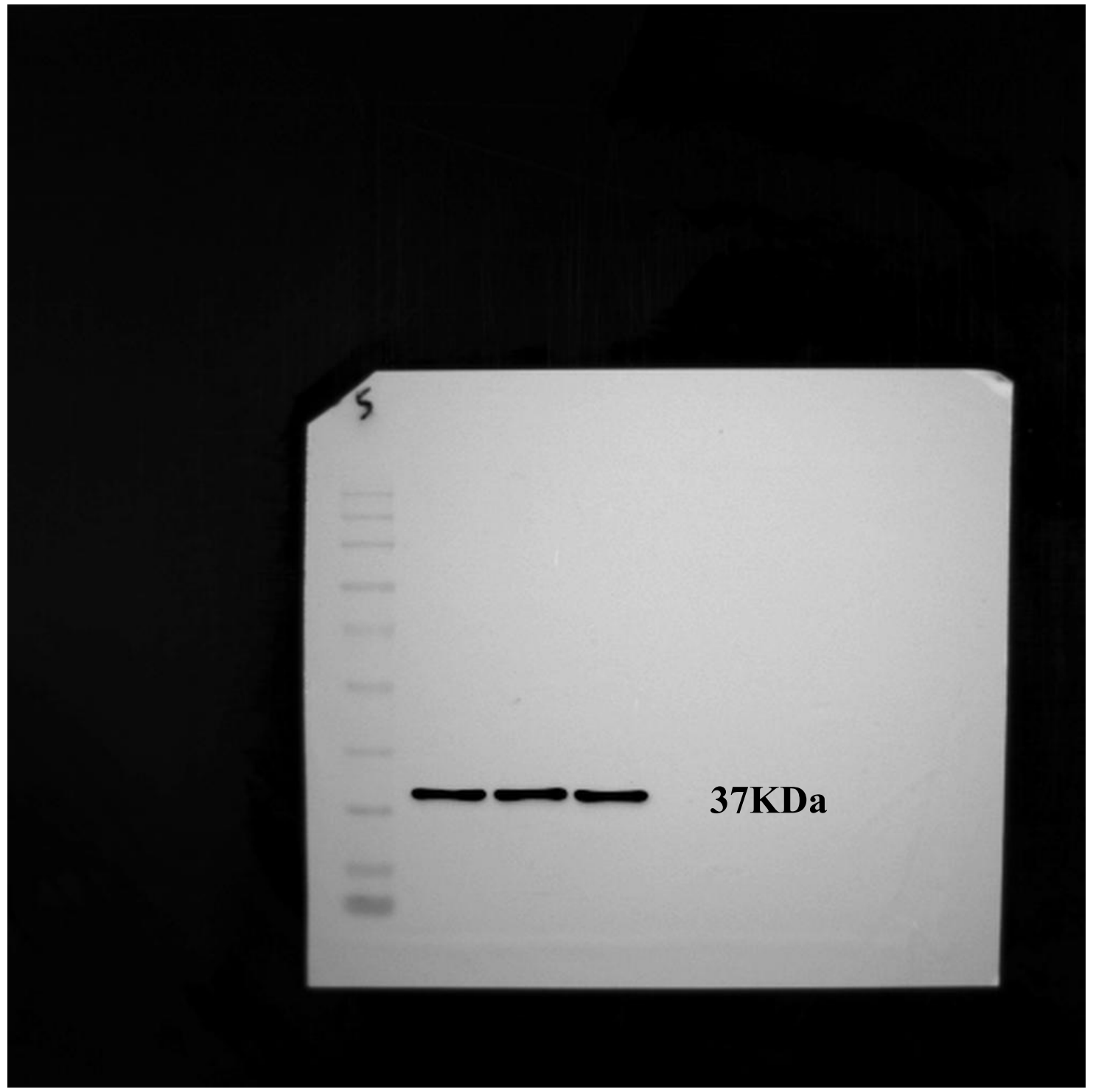

Target 4: mTOR (289 kDa) +Loading Control GAPDH (37 kDa)
(Corresponding to **Figure 6A**)

Full spliced membrane

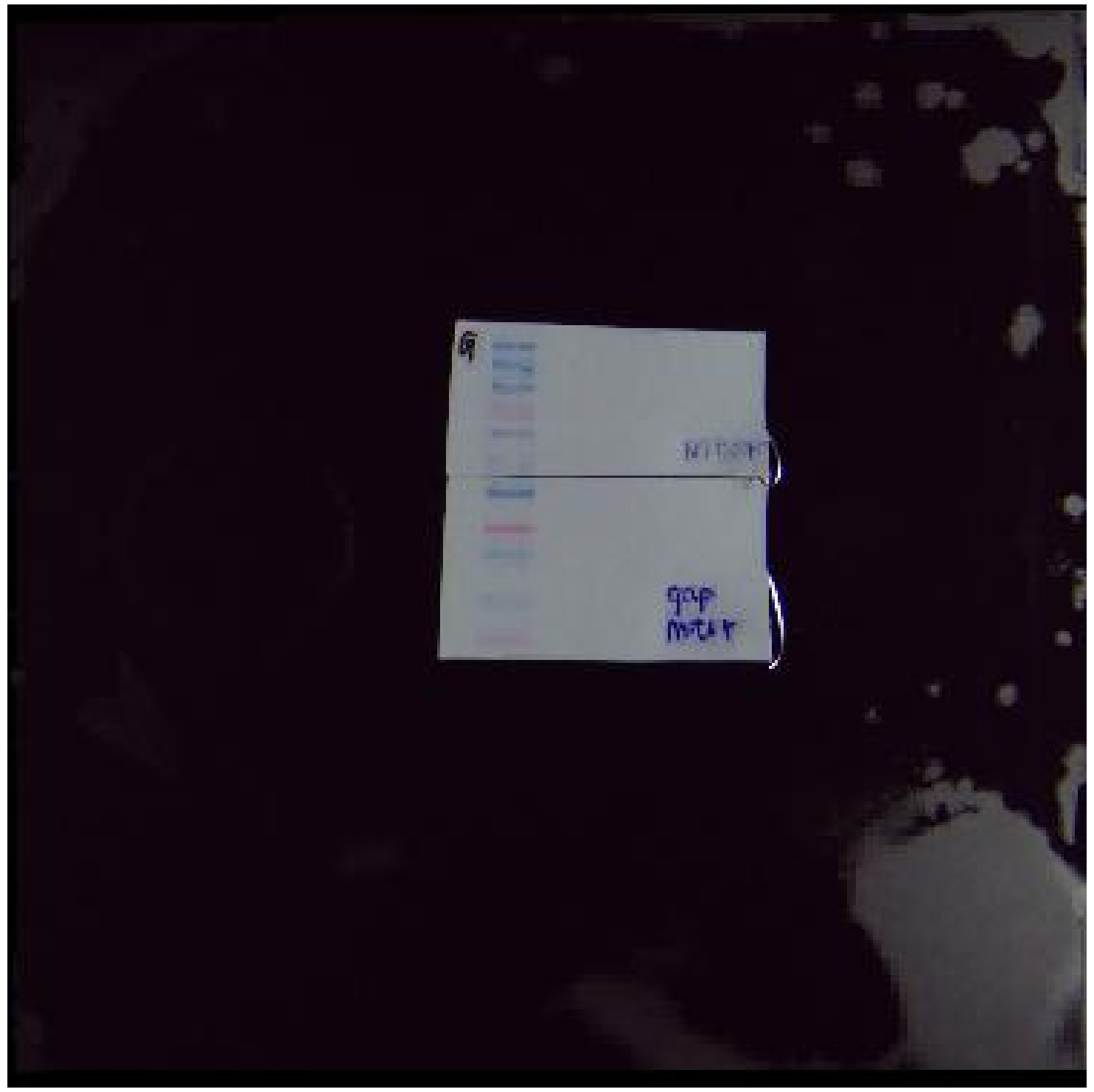

mTOR ~289 kDa

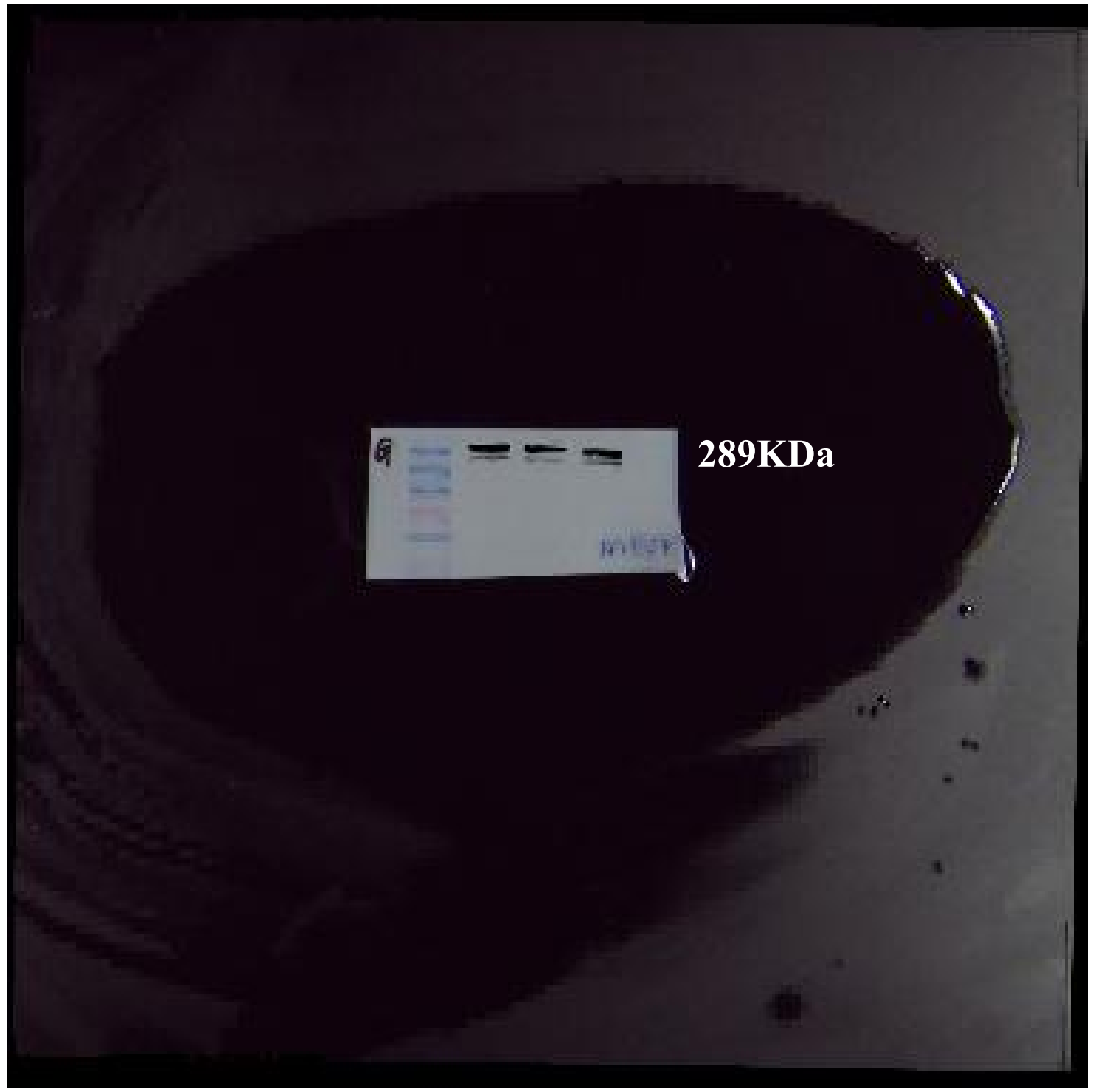

GAPDH ~37 kDa

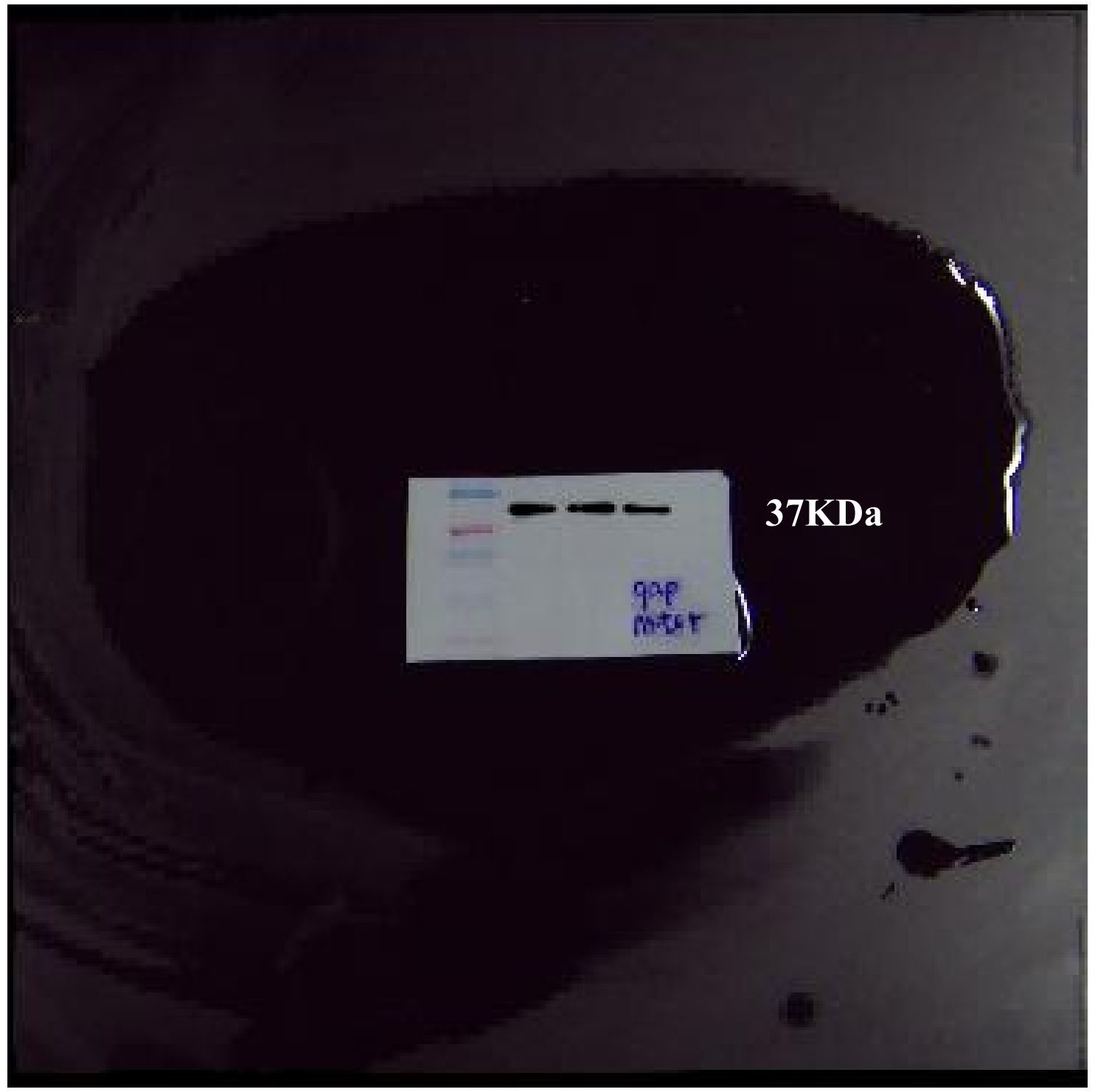

Target 5: p-GSK3β (47 kDa) + Loading Control HSP90 (85 kDa)
(Corresponding **to Figure 6D**)

HSP90 ~85kDa

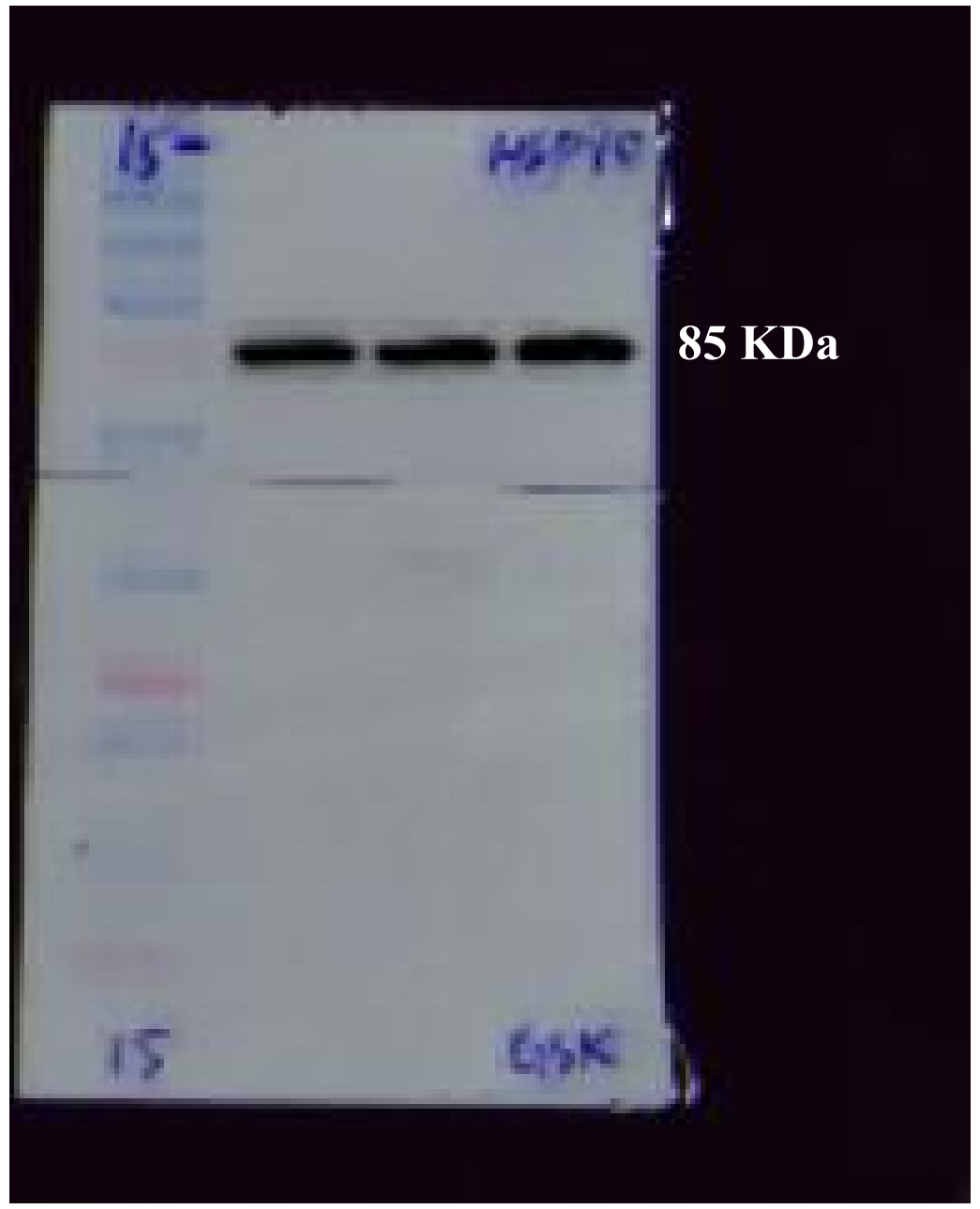

p-GSK3β ~47 kDa

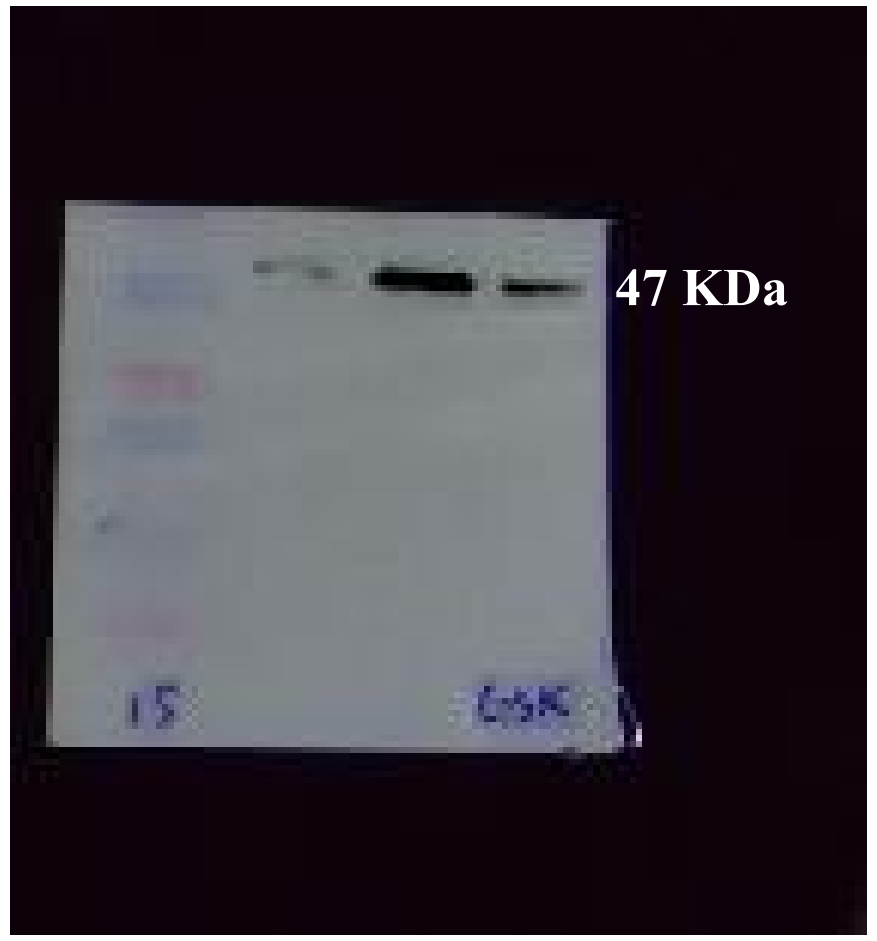

Target 6: GSK3β (47 kDa) + Loading Control HSP90 (85 kDa)
(Corresponding to **Figure 6D**)

HSP90 ~85kDa

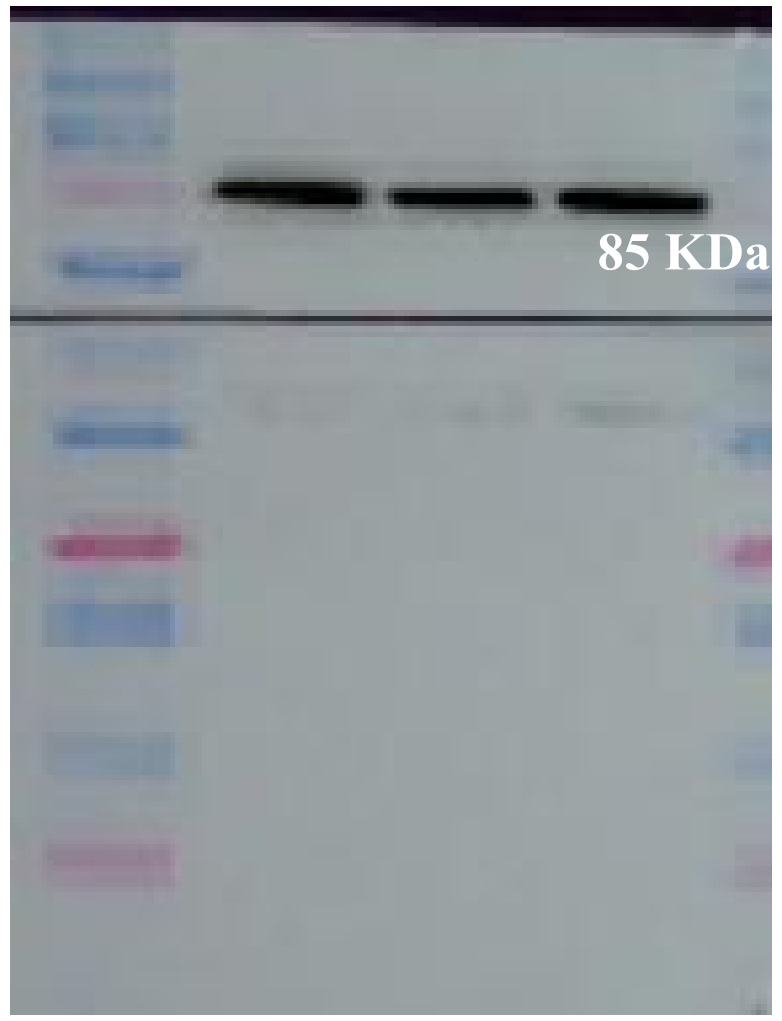

GSK3β ~47 kDa

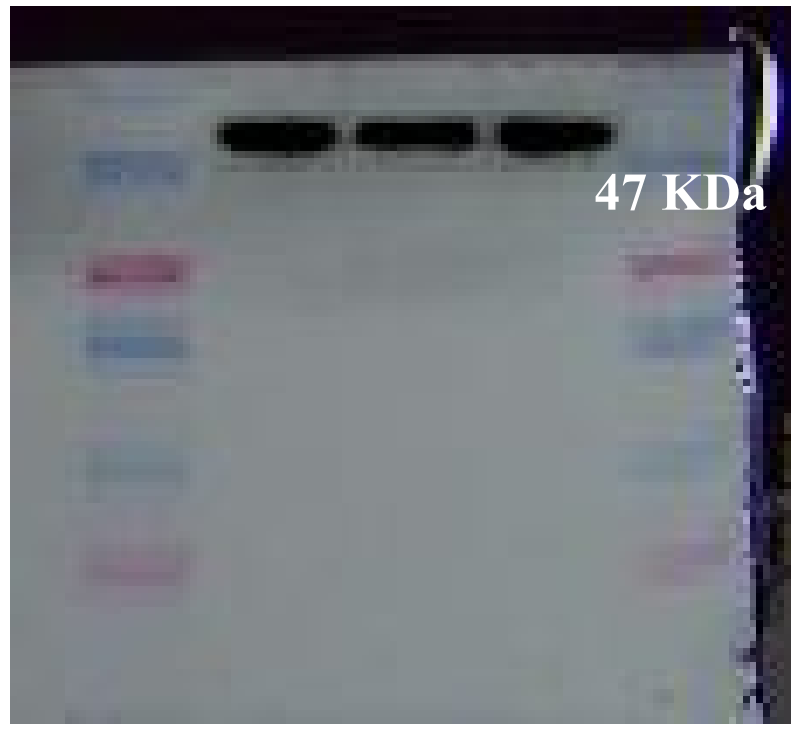

Target 7: p-4EBP1 (19 kDa) + Loading Control Tubulin(55 kDa)
(Corresponding to **Figure 6G**)

Tubulin~55 kDa

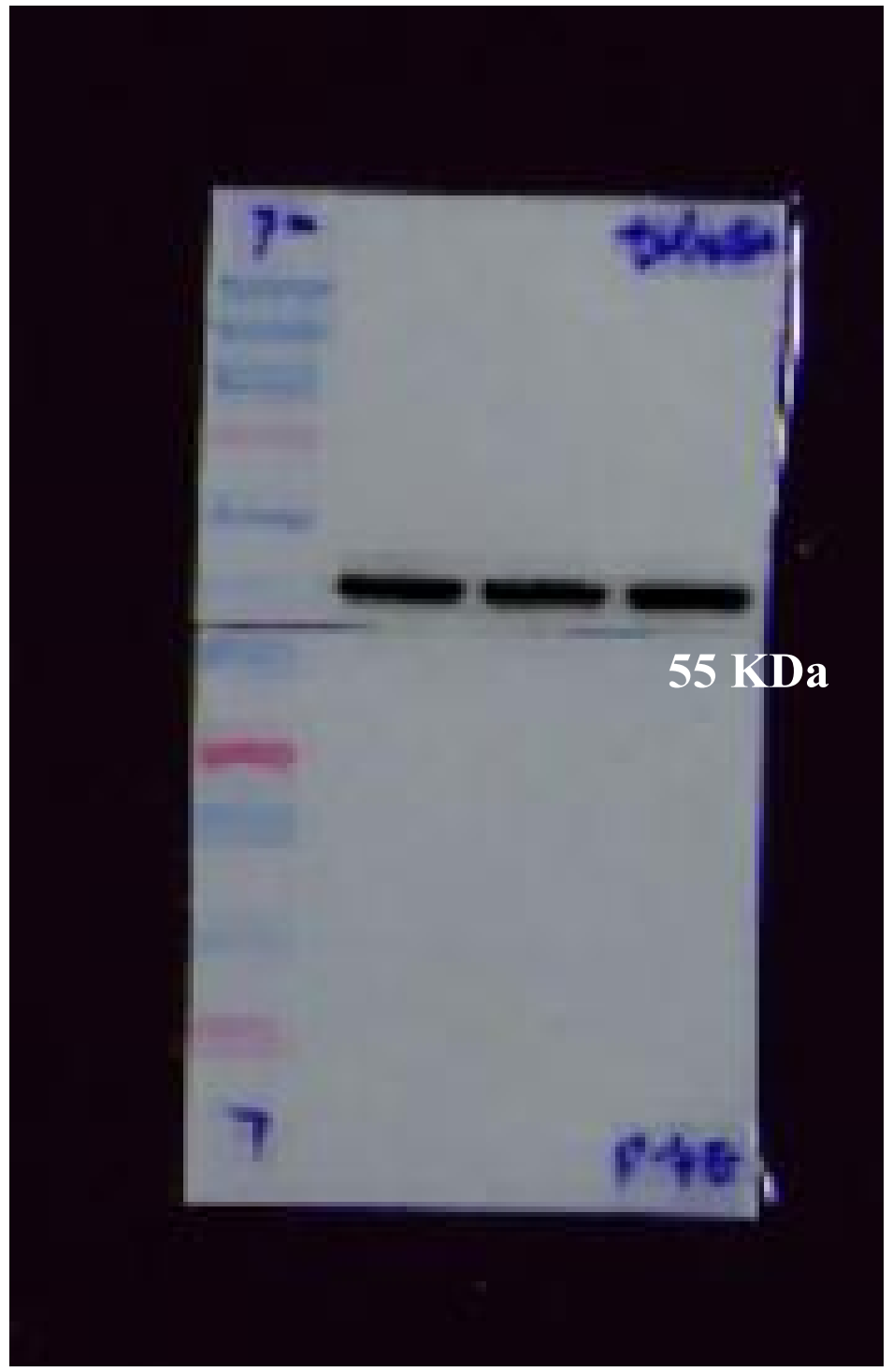

p-4EBP1 ~ 19 kDa

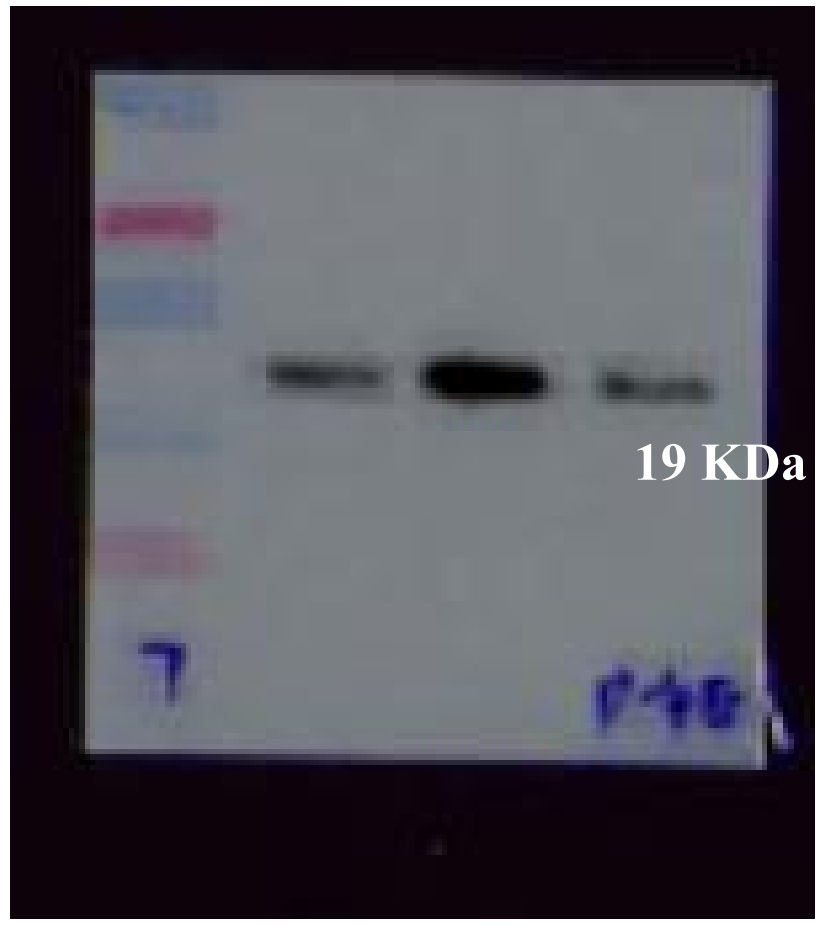

Target 8: 4EBP1 (19 kDa) +Loading Control Tubulin(55 kDa)
(Corresponding to **Figure 6G**)

Tubulin~55kDa

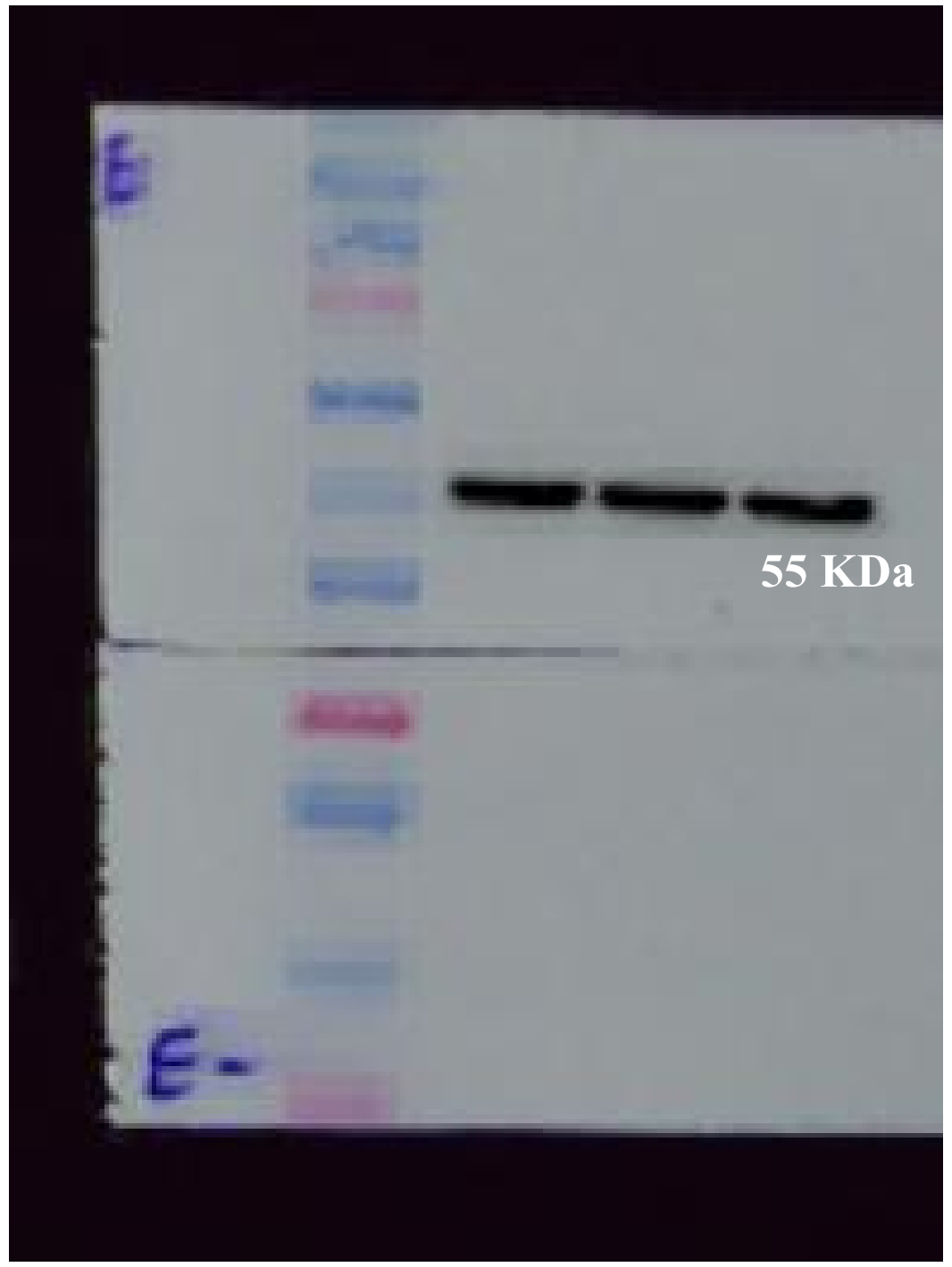

4EBP1 ~ 19 kDa

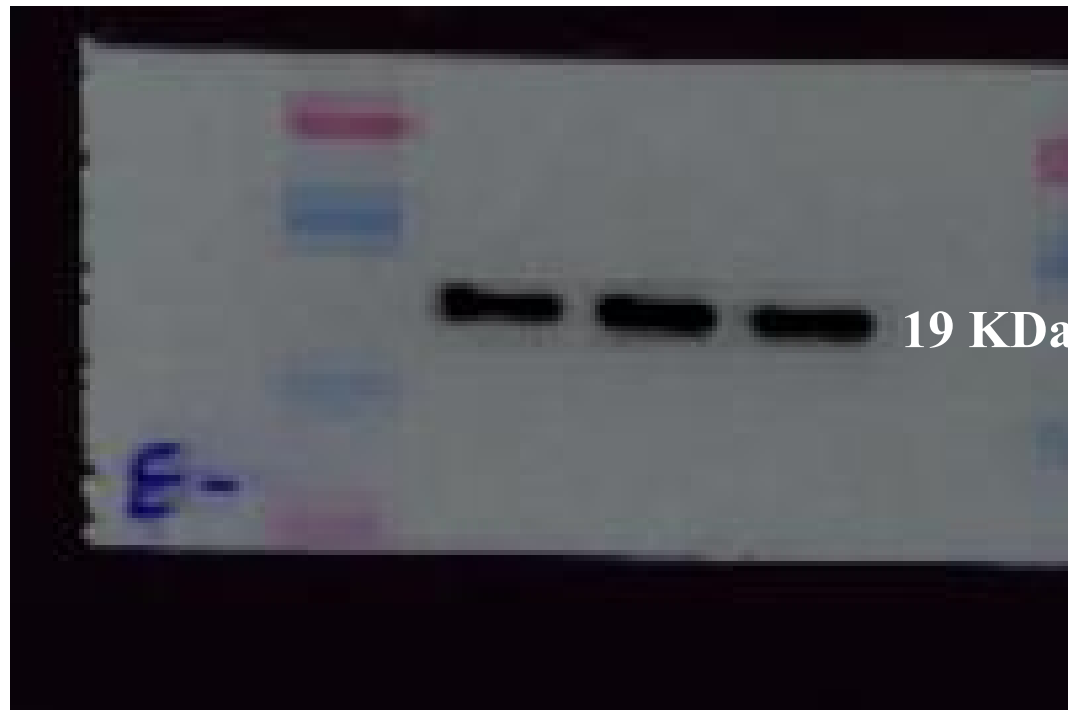

Target 9:PSD95 (90 kDa) + Loading Control GAPDH (37 kDa)
(Corresponding to **Figure 6J**)

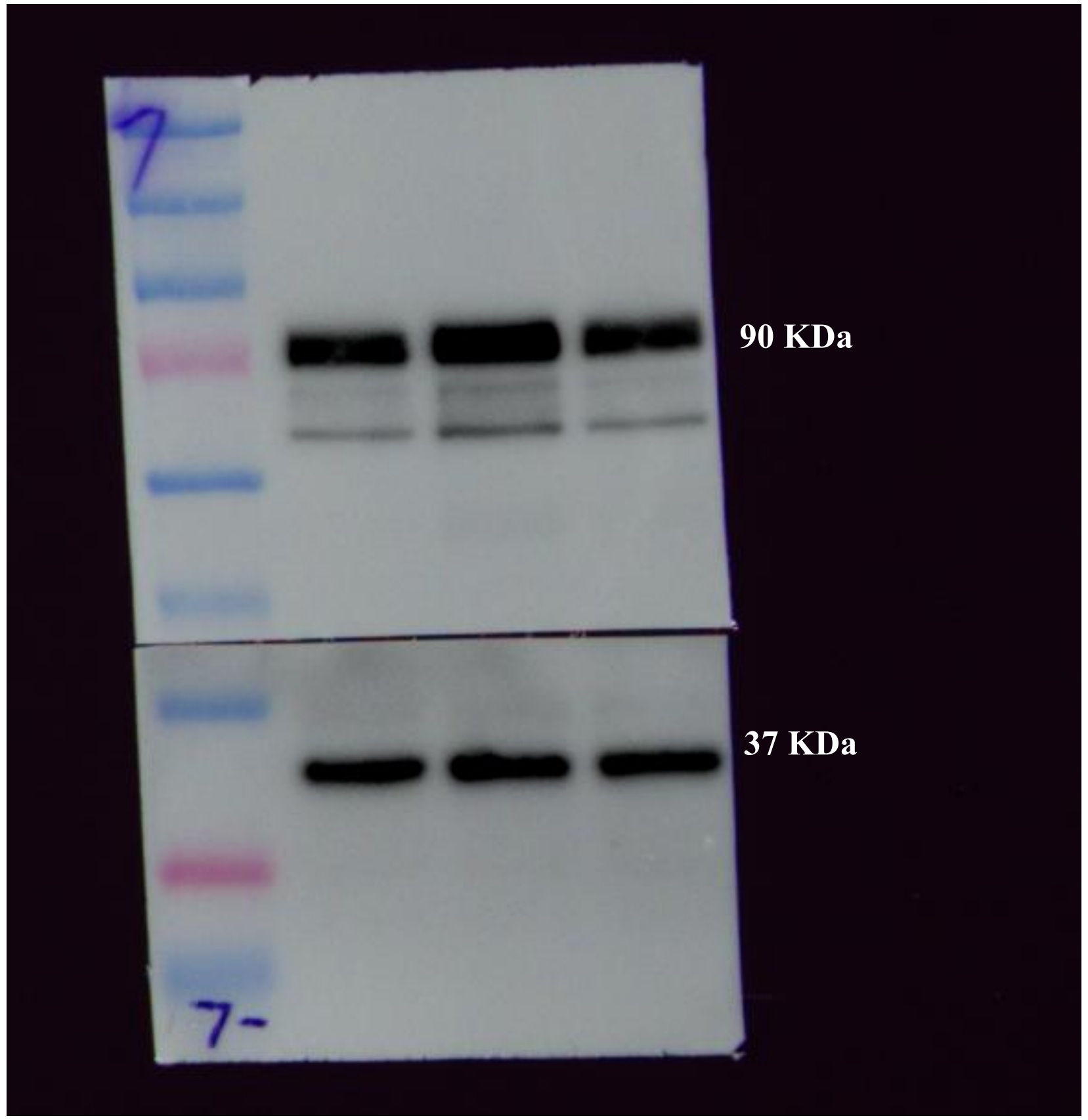

Target 10:GluA1 (100 kDa) + Loading Control GAPDH (37 kDa)
(Corresponding to **Figure 6L**)

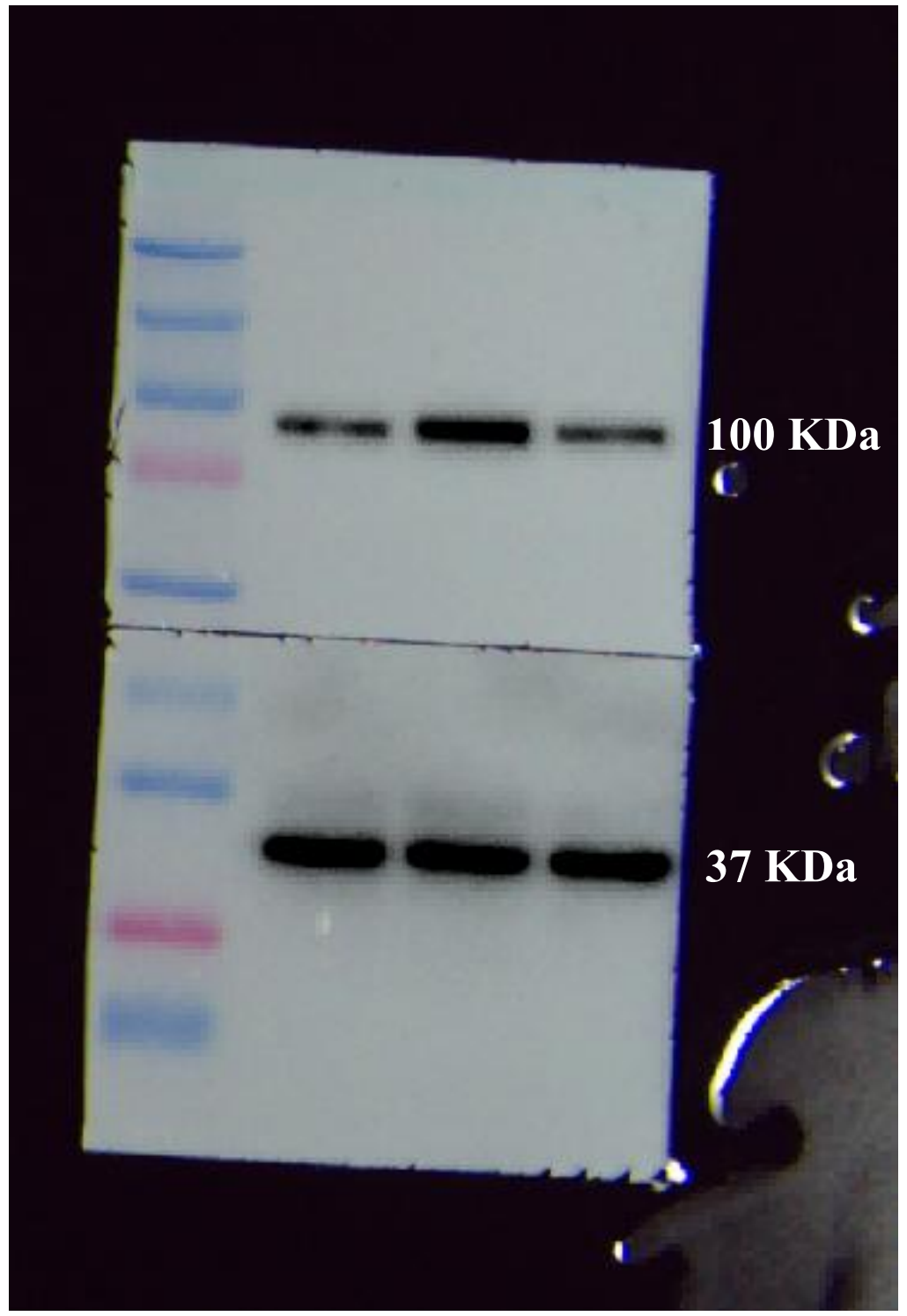

Target 11: CamkIIα (55 kDa) + Loading Control GAPDH (37 kDa)
(Corresponding **to Figure 6N)**

CamkIIα ~55 kDa

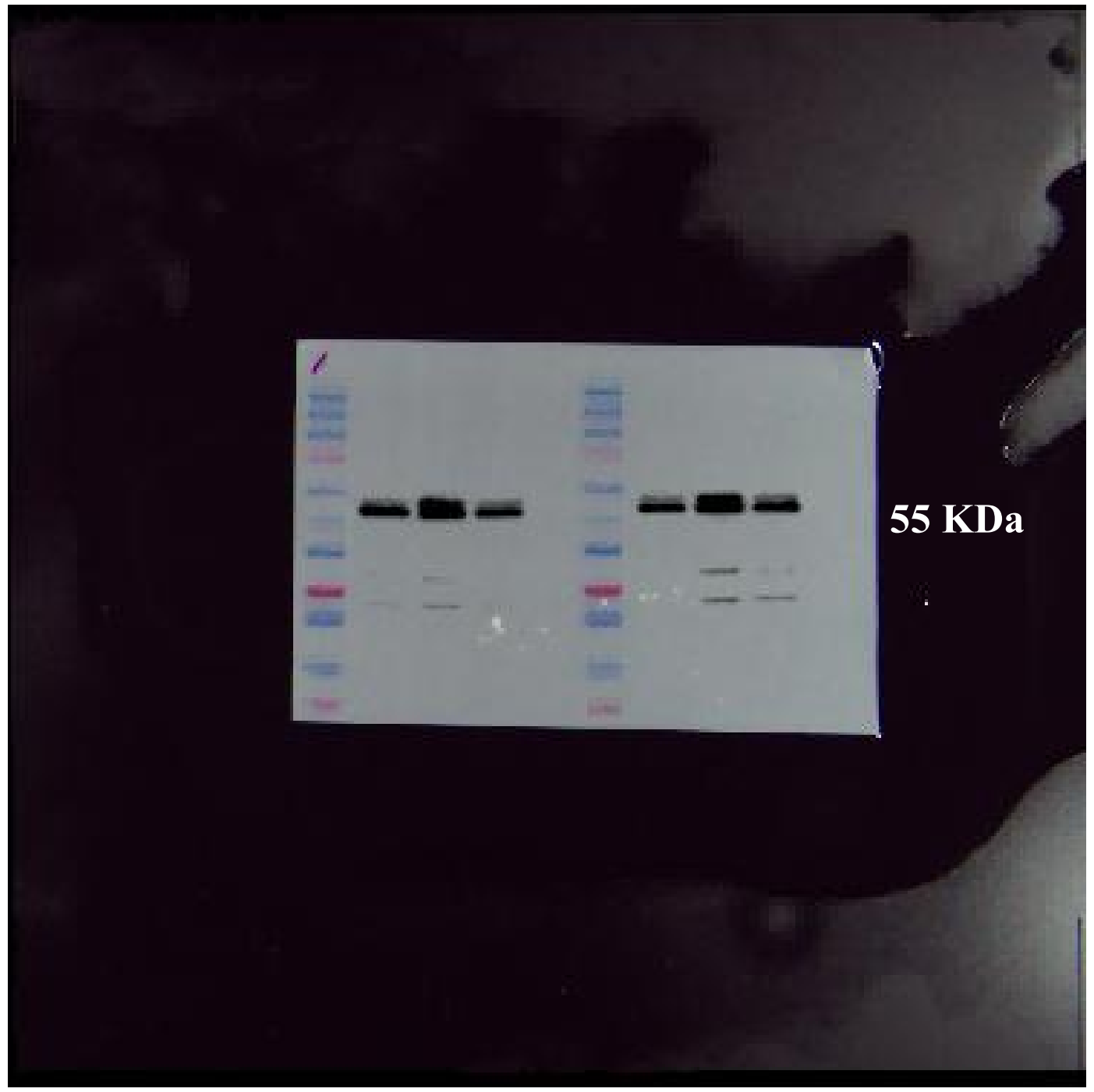

GAPDH ~37 kDa

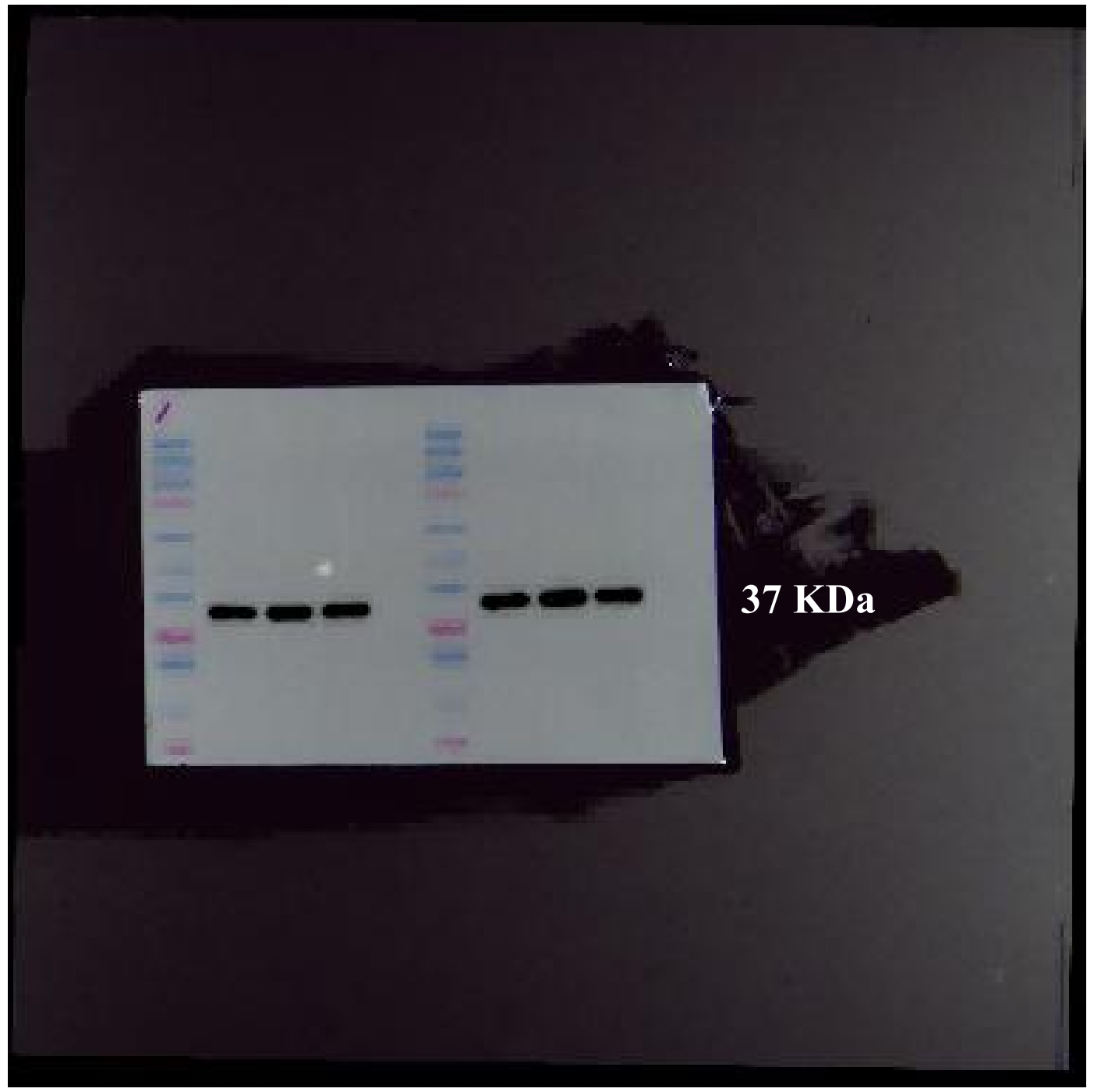

Target 12:GAD67 (67 kDa) + Loading Control GAPDH (37kDa)
(Corresponding **to Figure 6P**)

GAD67 ~67 kDa

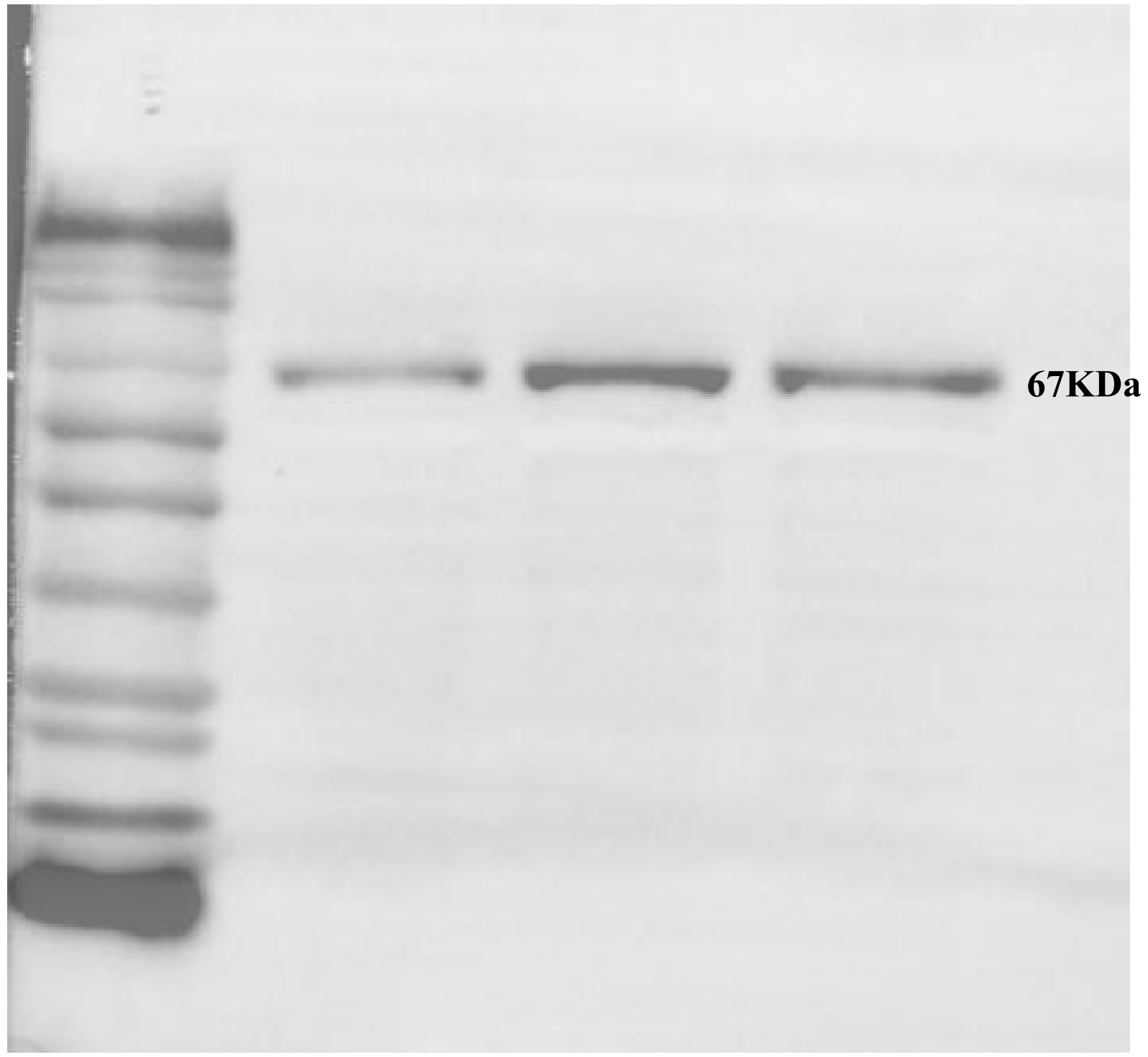

GAPDH ~37 kDa

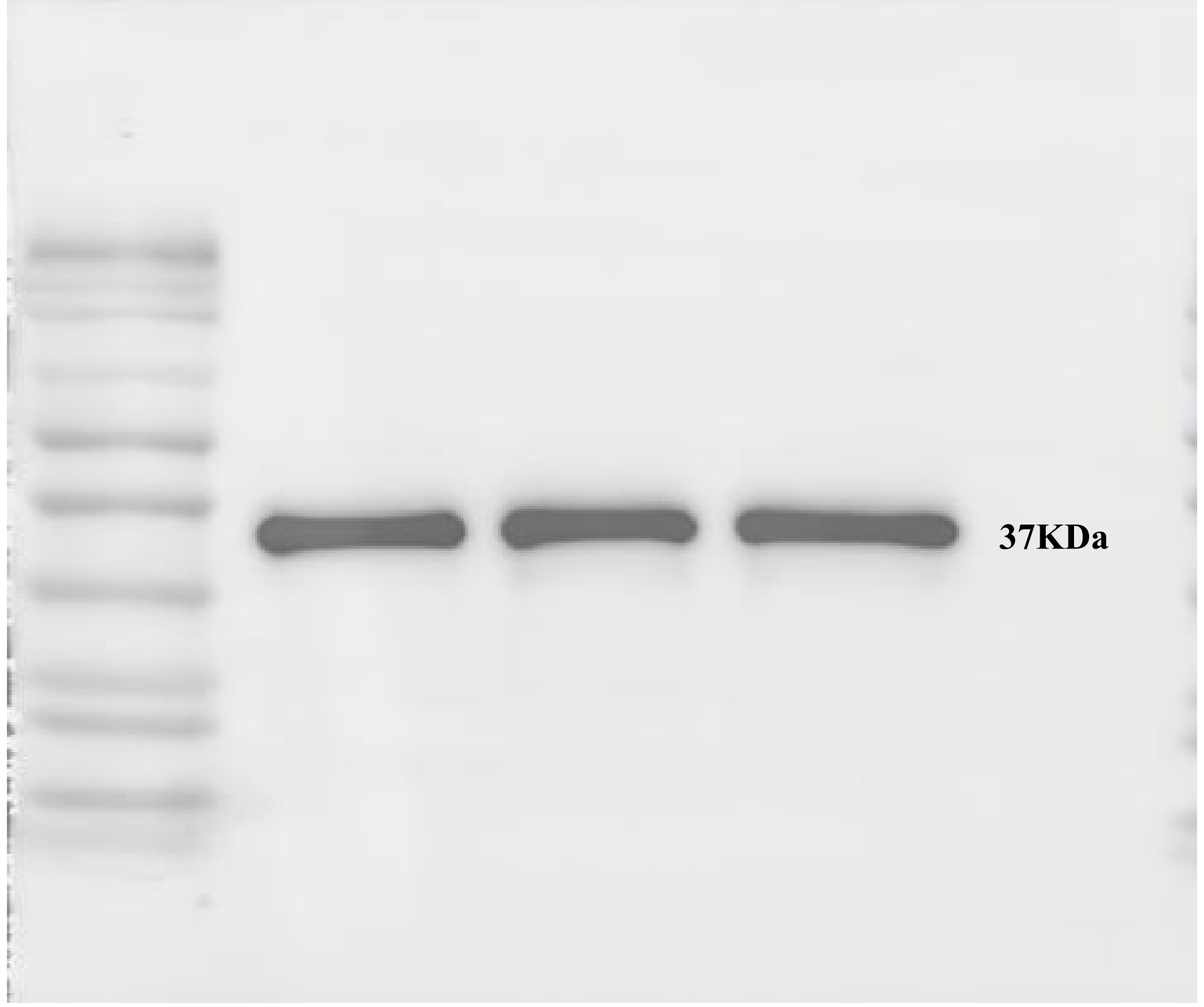

Supplement: Supplementary file 2 [file DataSheet2.pdf]
